# Improving extinction projections across scales and habitats using the countryside species-area relationship

**DOI:** 10.1038/s41598-017-13059-y

**Published:** 2017-10-10

**Authors:** Inês Santos Martins, Henrique Miguel Pereira

**Affiliations:** 1 German Centre for Integrative Biodiversity Research (iDiv) Halle-Jena-Leipzig, Deutscher Platz 5e, 04103 Leipzig, Germany; 2 Institute of Biology, Martin Luther University Halle-Wittenberg, Am Kirchtor 1, 06108 Halle (Saale), Germany; 3 Infraestruturas de Portugal Biodiversity Chair, CiBiO/InBIO, Universidade do Porto, 4485-661 Vairão, Portugal

## Abstract

The species-area relationship (SAR) has been often used to project species extinctions as a consequence of habitat loss. However, recent studies have suggested that the SAR may overestimate species extinctions, at least in the short-term. We argue that the main reason for this overestimation is that the classic SAR ignores the persistence of species in human-modified habitats. We use data collected worldwide to analyse what is the fraction of bird and plant species that remain in different human-modified habitats at the local scale after full habitat conversion. We observe that both taxa have consistent responses to the different land-use types, with strongest reductions in species richness in cropland across the globe, and in pasture in the tropics. We show that the results from these studies cannot be linearly scaled from plots to large regions, as this again overestimates the impacts of land-use change on biodiversity. The countryside SAR provides a unifying framework to incorporate both the effect of species persistence in the landscape matrix and the non-linear response of the proportion of species extinctions to sampling area, generating more realistic projections of biodiversity loss.

## Introduction

Globally, habitat loss and habitat degradation are identified as the main current threats to vertebrates, affecting more than 80% of globally threatened mammals, birds, amphibians and plants^1^. Assessing the response of species to different scenarios of land-use has become essential to predict patterns of species extinction and guide conservation actions^2^. Species-area relationship (SAR) models have been often at the heart of such assessments, projecting species extinctions as a consequence of loss of area of native habitat^3–5^.

The SAR is one of the oldest known patterns in ecology and has been studied in a wide variety of systems and scales^6^. Although a range of functions have been proposed to model the SAR, a power function relating the number of species *S* with the area of habitat *A*, $S(A) \sim {A}^{z}$, is most commonly used to assess species extinctions after habitat loss. Therefore, if an area *a* of the original native habitat is converted to human-modified habitat, the fraction of species that is predicted to go extinct (ε) is given by^7^

1 $$\varepsilon (a)=\frac{S(A)-S(A-a)}{S(A)}=1-{(\frac{A-a}{A})}^{z},$$

where *z* is a constant indicating the rate at which species richness increases with area.

Despite its widespread use, this technique has a number of limitations, leading to a mismatch between predicted extinctions and recorded extinctions^8,9^. Some studies have pointed that extinction estimates generated using the SAR are often greater than those from empirical data (e.g.^10,11^), while others argue that the SAR may actually underestimate species extinctions in the long run, particularly in highly fragmented landscapes (e.g.^12,13^). This debate is not yet settled as there are still unresolved issues. One issue is the extinction debt, i.e. the difference between the immediate extinctions of the species restricted to the area of lost habitat and the future extinctions of species which cannot persist in the long-term on the area of remaining habitat^7,14^. Some studies have argued that these two distinct extinction processes are described by different types of SAR, with the latter having a steeper slope^6,7,15^. Others have argued that the SAR can only be used to estimate immediate extinctions^11,16^.

Another issue, that we believe to be even more prevalent, is that many species are able to persist in human-modified habitats. In contrast, the classic SAR assumes that human-modified areas (e.g. agricultural lands) are completely hostile to biodiversity^7,17^. Some recent global biodiversity models address this problem by estimating extinctions based on empirical studies of local species richness response to habitat conversion^18,19^. However, they do not account for how the impacts of land-use change on biodiversity may vary non-linearly with the spatial grain of analysis^20^.

This third issue has not been appreciated until now and is a distinct conceptual problem from the well-known non-linear relationship between species extinctions and proportion of remaining habitat. For instance, according to the classic SAR (equation (1)), when 90% of the habitat is lost in a landscape, and assuming a z of 0.20, only 37% of the species are lost (global extinctions). However, one may ask if the same proportion of species go extinct in a small plot in the landscape (local extinctions).

The issue of extinction debt has been discussed in many papers (e.g.^21–23^) and we will not revisit it here. Instead, here we discuss how the countryside species-area-relationship provides a framework to project short-term species extinctions that take into account species persistence in the matrix and the non-linearity of species extinctions with sampling scale. Short-term projections may underestimate the long-term consequences of habitat loss but are perhaps more consistent with policy relevant time scales.

## The countryside species-area relationship

The classic SAR only captures the species richness response to changes in native habitat area, overlooking the diversity of species responses to changes in habitat composition. In order to address this problem Pereira and Daily^24^ proposed the countryside SAR. Although there have been other SAR models trying to account for the response of biodiversity to different habitat types^10,25,26^, the countryside SAR model is the only one that accounts for the differential use of habitats by different species groups. In the countryside SAR, the richness of each species group *i*, *S*_*i*_, is given by a function of the area of each habitat *j* in the landscape,

2 $${S}_{i}({A}_{1},{A}_{2},\ldots ,{A}_{n})=\,{c}_{i}{(\sum _{j=1}^{n}{h}_{ij}{A}_{j})}^{z},$$

where *n* is the number of habitat types, *h*_*ij*_ is the affinity of the species group *i* to habitat *j*, *A*_*j*_ the area of habitat *j*, and *c*_*i*_ measures the relative local abundance of each species group *i*. Then, the total number of species in the landscape, *S*, is given by the sum of all species groups. For simplicity, we use a power function to describe the countryside SAR (equation (2)) as this is the model typically used to predict species extinctions by area reduction, but whether other functions better describe the shape of the countryside SAR at different sampling scales is still an open question^27–29^.

Consider a single functional group (i.e. dropping the subscript *i* in equation (2)) and only two habitats, native (*j* = 1) and modified habitat (*j* = 2). Then, if an area *a* of a landscape of size *A* is converted, the fraction of species extinctions is

3 $$\varepsilon (a)=\frac{S(A,0)-S(A-a,a)}{S(A,0)}=1-\,{(\frac{{h}_{1}(A-a)+{h}_{2}a}{{h}_{1}A})}^{z}.$$

The countryside SAR predicts that some species always remain in the landscape (as long as ${h}_{2} > 0$). The proportion of species remaining will depend on their affinity to the human-modified habitats, *h*_2_. The countryside SAR describes the relationship between species richness and habitat area better than the classic SAR, both at local^28,30^ and regional scales^31^, and it also projects species extinctions more accurately^32^.

## Estimating the affinity of biodiversity to human-modified landscapes

A key parameter of the countryside SAR is the affinity of species to each habitat, *h*_*j*_. Affinity values can be estimated from local data studies (i.e. studies carried out at plot size scale). Let *σ*_*j*_ represent the sensitivity^10^ of species to the full conversion of native habitat into the modified habitat *j*, i.e. the proportion of species disappearing at the plot-scale in modified habitats:

4 $${\sigma }_{j}=1-\frac{S(j)}{S(1)},$$

where *S*(1) and *S*(*j*) represent species richness at the plot scale in the native and modified habitat of type *j*, respectively. Note that ${\sigma }_{j}$ equals ε (equation (3)) at the plot scale when the native habitat is fully converted to habitat *j* (i.e. *a* = *A*). It can be shown that ${h}_{j}={(1-{\sigma }_{j})}^{1/z}$ ^32^, since affinities and sensitivities are related (see Supplementary Note). A fully hostile modified habitat where all species go extinct corresponds to a sensitivity of one, while a fully hospitable modified habitat corresponds to a sensitivity of zero. It is also possible to have a negative sensitivity when the modified habitat has higher species richness than the native habitat (e.g. exotic species colonize the modified habitat).

**Figure 1:**
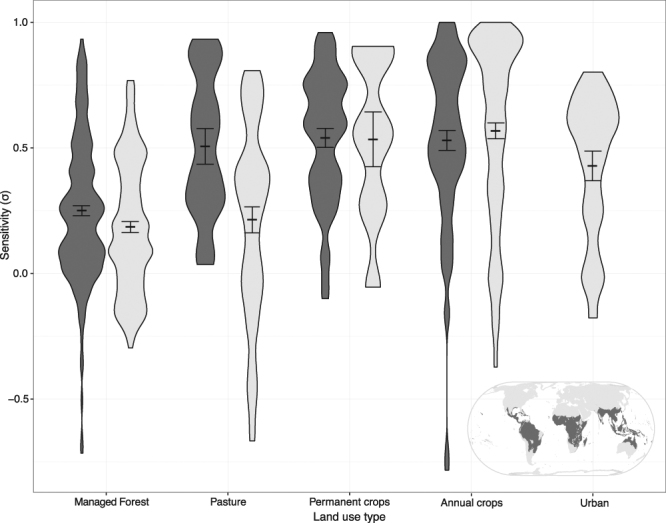
Local scale sensitivity (σ) of species in tropical (dark-grey; N = 355) and temperate (light-grey; N = 375) regions to the different human-modified habitats. The width and length of the polygons indicates, respectively, the density and range of the data. Error bars indicate standard errors. Inset map was created based on WWF terrestrial ecoregions^47^ in order to highlight the two distinct climate regions using ArcGIS 10.2.1 software^48^.

We examined the distribution of sensitivities of birds and plants to habitat conversion into different land-use types in two distinct climate regions (Fig. 1) and estimated the corresponding habitat affinities for the countryside SAR. For such analysis we used two previously published databases of local studies from across the globe where *S*(1) and *S*(*j*) were reported^33,34^ (see Supplementary Tables S1 and S2 and Methods). None of the taxon shows complete sensitivity to any of the transformed habitats (i.e. *σ* *=* 1). On average species respond better to the presence of managed forest and pastures than to the presence of crops, with some studies even reporting beneficial impacts (i.e. σ < 0). A three-way analysis of variance and an effect size analysis (see Supplementary Fig. S1) showed that while the effect of land-use was significant (*η* ^2^ = 0.20, F_4,712_ = 46.58, *p* < 0.001), the effect of taxon was non-significant. The interaction between these variables was also significant although small in effect (taxon × land-use type: *η* ^2^ = 0.02, *F*_4,712_ = 3.87, *p* < 0.01). In addition, species show similar sensitivities across the globe, but for some land-use types, the sensitivity may vary between tropical and temperate regions, as in the case of pastures (Fig. 1).

## Scaling biodiversity response to habitat conversion from the local level to the regional scale

The next problem is how to scale the response of biodiversity to land-use change from the local scale, which was the scale of the studies used to estimate these sensitivities, to the larger scales at which we often want to make projections. While one expects, based on the classic SAR (equation (1)), that species extinctions scale non-linearly with area of habitat loss, what happens to species extinctions as one changes the scale of analysis (i.e. sampling grain) has not been assessed until now.

**Figure 2:**
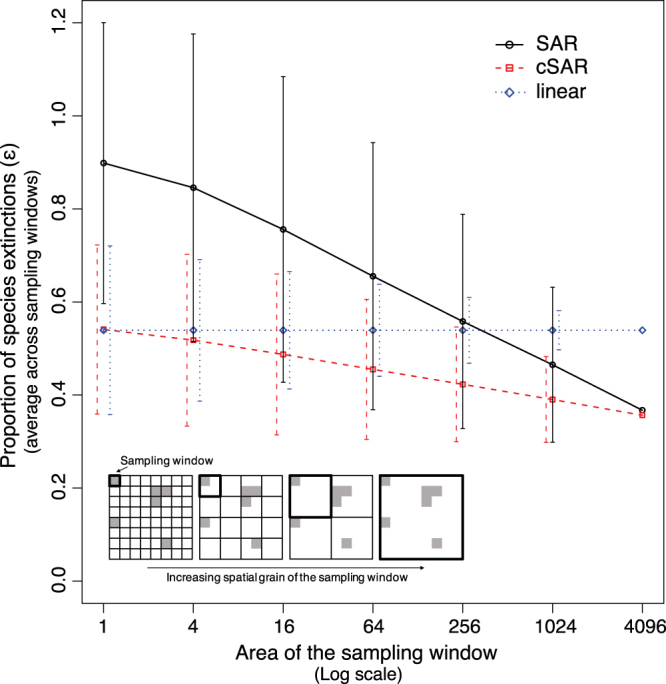
Proportion of species extinctions (ε) in the simulated landscape after 90% habitat conversion given by the linear, classic SAR and the countryside SAR. Points corresponds to the average number of species extinctions (across 1000 simulations) calculatsed in all sampling windows of a given sampling scale (a natural log transformation was applied to the area of the sampling window). Inset illustrates the nested sampling, with white squares corresponding to human-modified habitat and grey squares to the native habitat. For all models, *z* = 0.2, with, *h*_1_ = 1 for the native habitat and *h*_*2*_ = 0.01 for the modified habitat. Error bars indicate for each model, the standard deviation of the fraction of species remaining at a given sample grain.

Consider a landscape represented by a grid where each cell can be either native habitat or human-modified habitat (Fig. 2 inset, Methods). One can sample this landscape at a given window size, Ω and calculate the average proportion of species extinctions across all sample windows as (Supplementary Fig. S2),

5 $$\overline{\varepsilon ({\rm{\Omega }})}=\frac{{\sum }_{k}\varepsilon ({a}_{k}^{{\rm{\Omega }}})}{N({\rm{\Omega }})},$$

where ${a}_{k}^{{\rm{\Omega }}}$ is the area of human-modified habitat in the window *k* of size Ω, and *N*(Ω) is the total number of windows of size Ω in the landscape. In order to calculate the species extinctions in each sampling window of the landscape, $\varepsilon ({a}_{k}^{{\rm{\Omega }}})$, we used the countryside SAR (equation (3)), the classic SAR (equation (1)), and a linear model (countryside SAR with *z* = 1). The linear model assumes that the fraction of extinctions is directly proportional to the amount of habitat-modified times the species sensitivity ($\varepsilon (a)=\frac{a\cdot \sigma }{A}$, see Methods).

Using the linear model, the fraction of extinctions is constant across scales but is scale dependent for the countryside and classic SAR: the mean proportion of species extinctions decreases with increasing sampling scale for the two SAR models (Fig. 2). This suggests that the linear model overestimates the proportion of species going extinct at large scales. That is, the sensitivity of a taxonomic group to habitat change cannot be linearly extrapolated for scales other than those at which the study was conducted.

For a small sampling grain, the mean proportion of species extinctions estimated with the classic SAR approaches the proportion of human-modified habitat in the landscape (90% in Fig. 2, 50% and 10% in Supplementary Fig. S3). This proportion of extinctions is much higher than what is known to happen from the field studies at the local scale (linear model). This happens because the classic SAR assumes that the human-modified habitat is completely inhospitable (e.g. when 90% of the habitat is converted, local species richness becomes zero in 90% of the sites).

In contrast, in the countryside SAR matches the results from the field studies at the local scale (the linear model) and projects lower extinction rates at larger scales due to the non-linear relationship between habitat area and species richness (Fig. 2). This pattern occurs independently of the amount of native habitat remaining in the landscape, of the rate at which species richness increase with area (z value), of the sensitivity of species to human modified habitats, and of the degree of fragmentation of the landscape (Supplementary Figs S3 and S4). In addition, the variance of the proportion of species extinctions across sampling sites decreases with increasing sampling scale (Fig. 2). This decrease in the variance of species extinctions with scale is fastest with the linear model and slowest with the classic SAR.

## Discussion

SARs have been used to project biodiversity loss at regional to global scales but these projections ignore the persistence of species in human-modified landscapes. Here, we show that species response to habitat conversion vary significantly between land-use types, with some studies even reporting a positive response to human-modified habitats. We did not distinguish between specialist (i.e. occurring in only one habitat) and generalist species (i.e. occurring in more than on habitat), and while we can expect within taxon responses to vary among species functional groups (e.g. forest bird species, agriculture bird species), species are known to show dissimilar degrees of tolerance to habitat conversion, even among habitat specialists^35^. On average species respond better to the conversion of native habitat to managed forests and pastures than to cropland. Our finding corroborate those by Gibson *et al*.^36^ and Newbold *et al*.^19^ among others. Both studies found cropland areas to have some of the strongest global effects on biodiversity, although Newbold *et al*. identifies urban areas as the land-use with the highest effects on biodiversity loss. This is mostly due to a strong effect of land-use intensity in urban areas found by Newbold *et al*., where urban areas are particularly hostile to biodiversity when intensively used, but have the lowest effects on biodiversity when minimally used. The sensitivity of species to urban areas seems to vary highly with the degree of land-use intensity^19^.

Although we did not observe significant differences across taxa and region, we found that for some land-use types, the sensitivities may vary among plants and birds and between tropical and temperate regions. Birds species tend to be more sensitive to forest conversion into agriculture, whereas plants are more sensitive to burned forests and shaded plantations^36–38^. Furthermore, in some cases habitat conversion can lead to increase of richness of a taxon or a specific species group while decreasing the richness of another taxon or species group^39^ (e.g. when forest is converted to cropland, farmland bird species may increase their richness while forest bird species decrease theirs). Several studies have highlighted that tropical regions, especially South America and Southeast Asia, are particularly vulnerable to all forms of human impact^36^. We found species to have similar sensitivities to habitat conversion in tropical areas and temperate areas, with species responding differently only to particular habitats, such as pastures (i.e. species more sensitive to pastures in tropical areas). This could be a result of the recent and widespread expansion of pasture areas in the tropics, whereas in temperate regions, such areas have existed for millenia^40^.

Overall, the likelihood of species undergoing extinction following habitat loss will depend on their sensitivity to the modified habitat and the capacity of the modified habitats to support them^10,17,24,34^. Consequently, SAR-based extinction projections are only reliable for species with zero affinity for the human-modified habitats. Here, we have estimated habitat affinity values for a range of land-use types, which can then be used in conjunction with the countryside SAR to project biodiversity responses at scales larger than the plot scale for which field data was originally collected. The methodology used here to calculate habitat affinities can also be applied to other databases of biodiversity responses to land-use (e.g.^19^).

Empirical studies have suggested that as the spatial grain increases, the effect of land-use on biodiversity patterns tends to decrease^31^, which may lead to the signal of land-use change being difficult to detect at large spatial scales. At very small scale the habitats are homogenous and one either counts species entirely within native or entirely within a human-modified habitat. In contrast at larger scales, any sampling unit is a mixture of both habitats and the non-linear effects of the SAR come into play.

To our knowledge, this non-linearity of the response of biodiversity to land-use change across sampling scales (or grain of analysis) has not been theoretically analysed before. Recently, Keil *et al*.^20^ has shown that empirical extinction rates may vary with sampling area, using extinction data from plants and butterflies across the European continent and North America. Interestingly they showed that this variation may be non-monotonic with scale: number of extinctions at local and large scale may be similar, but lower or higher than the number of extinctions at intermediate scales, depending on whether the response curve is convex or concave. The number of extinctions is the product of the proportion of species going extinct with the number of species. We already knew that the number of species increases with sampling area, as predicted by the species-area relationship. If the proportion of species going extinct was constant with scale, this would result in a monotonic increase of the number of species going extinct with scale. Here we show that the proportion of species going extinct may decrease with increasing sampling scale. Therefore, our results are consistent with the findings of Keil *et al*.^20^ of a non-monotonic relationship between number of extinctions and sampling scale.

This non-linearity of proportion of species extinction with sampling scale calls for some caution when interpreting maps of impacts of land-use change coming out of models such as GLOBIO and PREDICTS^18,19^. Studies using these models often plot the reduction in species richness using grid cells of 50 km × 50 km or larger based on plot level responses to land-use change. Our analysis suggests that the reductions at those scales may be significantly smaller than those at the plot scale, and therefore these maps should be interpreted as the plot-scale mean reduction in species richness. These maps are therefore not comparable with, for instance, analysis of changes of atlas of species distribution collected at those scales.

Furthermore, our results suggest that caution should be used when interpreting species richness trends from local studies. Recent global meta-analysis have found no reduction in species richness over time in time series of community assemblage data^41,42^. Hill *et al*.^43^ used a simple narrative based model to show that there is a large variance in plot-based species richness and therefore it may be difficult to detect a decline in species richness, even when the decline is clear at the regional level. Similarly, we found that, when all the sites are aggregated in the analysis independently of whether habitat conversion has occurred or not, the variance in the proportion of species extinction estimates is very large. This detection problem is particularly exacerbated when the signal of species richness change is small due to only a small proportion of the habitats haven been converted.

The complexity of studying biodiversity change across scales and habitats should not be underestimated. It is empirically challenging and requires a solid theoretical background. Our study contributes to a deeper understanding of SAR models and their applicability when projecting species extinctions as a consequence of habitat loss. Finally, we demonstrate that the countryside species-area relationship^24^ provides a unifying framework to account both for the effects of species persistence on the matrix and for the non-linearity of biodiversity response with scale. Improved global analyses of biodiversity loss are needed, specially to better inform future conservation goals (e.g. post- 2020 Aichi Targets) and ongoing policy-driven assessments (e.g. IPBES Global Assessment). The extinction projections from the countryside SAR may be less catastrophic but they are certainly more realist and will ultimately allow for better decision-making.

## Methods

### Studies used to estimate the affinity of biodiversity to human-modified landscapes

Sensitivity values (σ) were taken from two previously published global databases^33,34^ of studies of biodiversity responses to human-modified landscapes. From within these databases, we selected studies that provided data on bird and plants species richness on both native habitat and at least one human-modified habitat. In several of these studies, data for multiple habitat types, locations and/or species groups were reported, which led to a total of 730 pairwise comparisons. The databases do not distinguish between specialist species or generalist species, as in most cases the number of species in a given habitat was the only data reported. The data was subset into five land-use classes based on the description of the habitat given in the source paper: annual crops, managed forest, permanent crops, pastures and urban; and two major biomes: tropical and temperate. For all studies (see Supplementary Table S1) the databases report the sensitivity of a taxonomic group to habitat *j*, *σ*_*j*_, as the difference between the plot scale species richness found in the modified habitat of type *j* and the species richness in the native habitat (see equation (4)). Habitat affinities can be directly derived from these sensitivities (see Supplementary Note for details).

### Models for scaling the biodiversity response to habitat conversion

To demonstrate and analyse the effect of sampling scale on extinction projections, we simulated 1000 spatially-explicit landscapes represented on a lattice of 64 × 64 grid cells (inset Fig. 2). We randomly created these landscapes by classifying 10% of the cells as native habitat. We examined the effect of fragmentation by generating landscapes with variable clustering: from a single native-habitat fragment with 410 cells to 410 fragments with one cell each. For each landscape, we calculated extinctions using increasing sampling windows of size Ω (i.e.$\,{\rm{\Omega }}=1\times 1,\,{\rm{\Omega }}=2\times 2,\,{\rm{\Omega }}=4\times 4,$ etc) until the size of the lattice was reached (i.e. 64 × 64). We used the classic SAR, countryside SAR and the linear model to project the proportion of species extinctions in each sampling window *k* of size Ω in the landscape,$\,\varepsilon ({a}_{k}^{{\rm{\Omega }}})$. Note that at the smallest sampling scale, sampling windows are only comprised of 1 cell (Ω = 1), so we calculate $\varepsilon (a)$ for each of the cells (i.e. $\varepsilon ({a}_{1}^{1}),\,\varepsilon ({a}_{2}^{1}),\ldots ,\varepsilon ({a}_{k}^{1})$) while at the largest sampling scale, the sampling window is comprised of all cells and $\varepsilon (a)$ is calculated for the entire landscape (i.e. $\varepsilon ({a}_{1}^{4096})$) (see Supplementary Fig. S2 for more details). Finally, at each sampling scale, the projections of each sampling window were averaged to obtain the overall proportion of species going extinct in the landscape (equation (5)). This procedure was repeated for each of the 1000 different spatially-explicit landscapes, and the resulting 1000 proportion of species extinctions curves for each model were averaged to produce Fig. 2.

Note that the linear model can be derived from equation (3) when z = 1,

6 $$\varepsilon (a)=\frac{a{(1-{h}_{2})}^{1}}{A}=\frac{a\cdot \sigma }{A}.$$

Therefore, using the linear model, $\overline{\varepsilon ({\rm{\Omega }})}\,\,$is constant across scales but depends on the scale for the countryside and classic SAR models (z < 1).

We used the highest mean sensitivity for a human-modified habitat from Fig. 1, ${\sigma }_{P.crops}=0.6$ (h_1_ = 0.01, see Supplementary Note and Supplementary Table S2) and a value of *z* = 0.20 for the classic SAR and countryside SAR models, as it is an intermediate value of the wide range of *z*’s reported in the literature for both plants and birds at this spatial scales^6,29,44,45^. In order to understand how the results from the three models change with different parameter values, we varied z from 0.1 to 0.3, ${\sigma }_{1}\,\,$from 0 to 0.75, and the amount of native habitat left in the landscape from 10% to 90% (see Supplementary Fig. S3). We also explore in Supplementary Fig. S4, how the different models will behave in landscapes with different degrees of fragmentation (spatial clustering). These operations were carried out using RStudio 1.0.44^46^, and the full R code is available on GitHub (https://github.com/ISMartinss/IEPAScaleHabitat).

### Data accessibility

The dataset supporting this article have been uploaded as part of the supplementary material (Supplementary Table S1). Complete R code used for the simulations can be download here: https://github.com/ISMartinss/IEPAScaleHabitat.

## Electronic supplementary material

Supplementary information
