# Supplementary material for: Improving extinction projections across scales and habitats using the countryside species-area relationship
Source: Sci Rep. 2017 Oct 10;7:12899. doi: 10.1038/s41598-017-13059-y (PMC5635007; doi:10.1038/s41598-017-13059-y)
Supplement: Supplementary file 1 — Supplementary information [file 41598_2017_13059_MOESM1_ESM.pdf]

Supplementary information for

Improving extinction projections across scales and habitats using the countryside species-area relationship

Inês S. Martins [1,2], Henrique M. Pereira [1,2,3]*

[1] German Centre for Integrative Biodiversity Research (iDiv), Halle-Jena-Leipzig, Deutscher Platz 5e, 04103 Leipzig, Germany

[2] Institute of Biology, Martin Luther University Halle-Wittenberg, Am Kirchtor 1, 06108 Halle (Saale), Germany

[3] Infraestruturas de Portugal-Biodiversidade, CIBIO/InBIO, Universidade do Porto, 4485-661 Vairão, Portugal

**E-mail: ines.martins@idiv.de*

Contents:

Note: Relation between sensitivities (σ) and affinities (h).

Figure S1: Effect size analysis.

Figure S2: Scheme exemplifying how the simulated landscapes were sampled.

Figure S3: Proportion of species extinctions in the simulated landscape after habitat conversion given by the linear, classic SAR and the countryside SAR.

Figure S4: Proportion of species extinctions in the simulated landscape after 90% habitat conversion given by the linear, classic SAR and the countryside SAR, when varying the degree of fragmentation of the landscape after habitat conversion.

Table S1: Local sensitivities (sigma) for each land use type.

Table S2: Average affinity values (h's) for the different habitats and regions of the world.

Supplementary Note

Here we show, in greater detail than in the main text, that sensitivities (σ) and affinities (h) are related. If one assumes full habitat conversion ($A - a = 0$ and $a = A$), and that species have maximum affinity for the native habitat, $h_1 = 1$, the affinity of a species group to the habitat of type j, can be derived with equation (3) in the main text:

$$\varepsilon(a) = \frac{S(A,0) - S(0,A)}{S(A,0)} = 1 - \left(\frac{h_j\, A}{A}\right)^z = 1 - h_j^z. \tag{1}$$

Knowing that, σ_j equals ε (equation (3)) at the plot scale when the native habitat is fully converted to habitat j (i.e. $a = A$):

$$\sigma_j = 1 - h_j^z. \tag{2}$$

Thus, it is straightforward to show that:

$$h_j = (1 - \sigma_j)^{1/z}\,. \tag{3}$$

Supplementary Figures

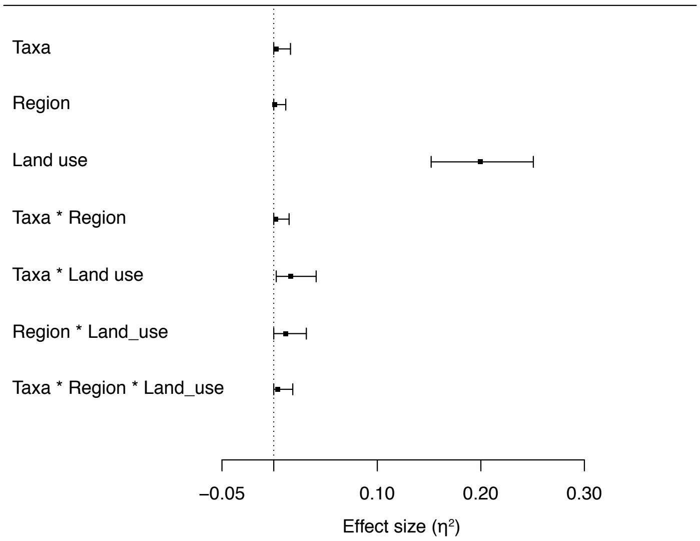

Supplementary Figure S1. Effect size (eta squared - η^2) and their confidence intervals (95% CIs) for the different categorical variables (i.e., taxa, region and land use) and their interactions analysed with a three-way ANOVA (N=730).

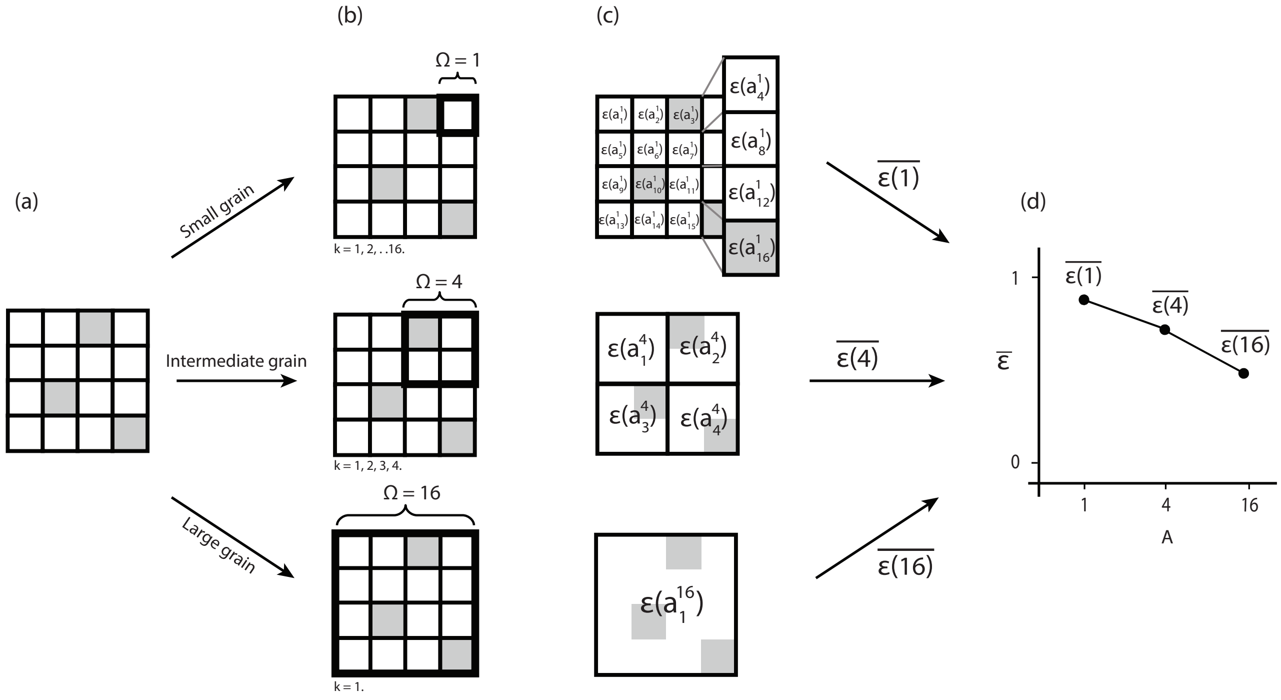

Supplementary Figure S2. Scheme exemplifying how the simulated landscapes were sampled to calculate the average proportion of species extinctions $\overline{\varepsilon(\Omega)}$, at each sampling grain, Ω. (**a**) potential configuration of a simulated landscape, illustrated for a 4 x 4 = 16 grid, with white squares corresponding to human-modified habitat and grey squares to the native habitat. (**b**) three different sampling grains: at the small sampling grain, sampling window *k* is only comprised of 1 cell ($\Omega = 1, N(\Omega) = 16$), at the intermediate sampling grain, sampling window k is comprised of 4 cells ($\Omega = 4, N(\Omega) = 4$) and at the large sampling grain, there is only one sampling window k and comprises all cells ($\Omega = 16, N(\Omega) = 1$).

(**c**) For a given model (see Methods), species extinctions, $\varepsilon(a_k^{\Omega})$, were calculated for each sampling window *k* of size Ω in the landscape (**d**) At each sampling grain, the projections of each sampling window were averaged to obtain the overall fraction of species going extinct in the landscape, where $\overline{\varepsilon(\Omega)} = \frac{\sum_k \varepsilon(a_k^{\Omega})}{N(\Omega)}$.

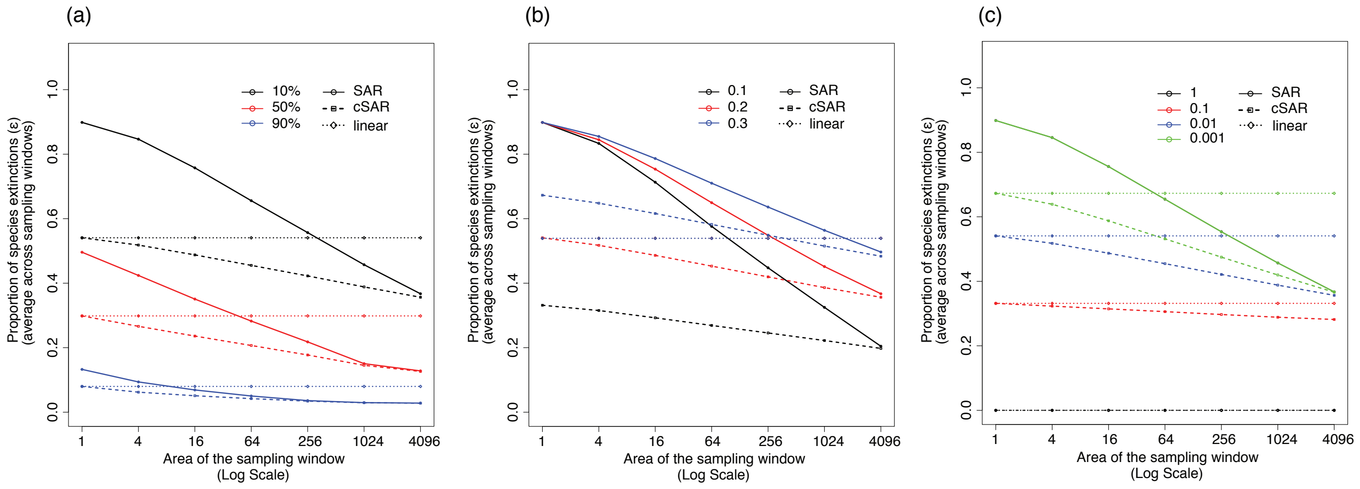

Supplementary Figure S3. Proportion of species extinctions (ε) in the simulated landscape after habitat conversion given by the linear, classic SAR and the countryside SAR. When varying **(a)** amount of native habitat left in the landscape (when z=0.2 and h_2=0.01), (**b)** the z-value (for 10% habitat remaining and h_2=0.01) and **(c)** species sensitivity for the modified habitat (for 10% habitat remaining and z=0.2). For all scenarios, h_1=1 for the native habitat. Points corresponds to the average number of species (across 1000 simulations) found in all sampling units of a given sample grain.

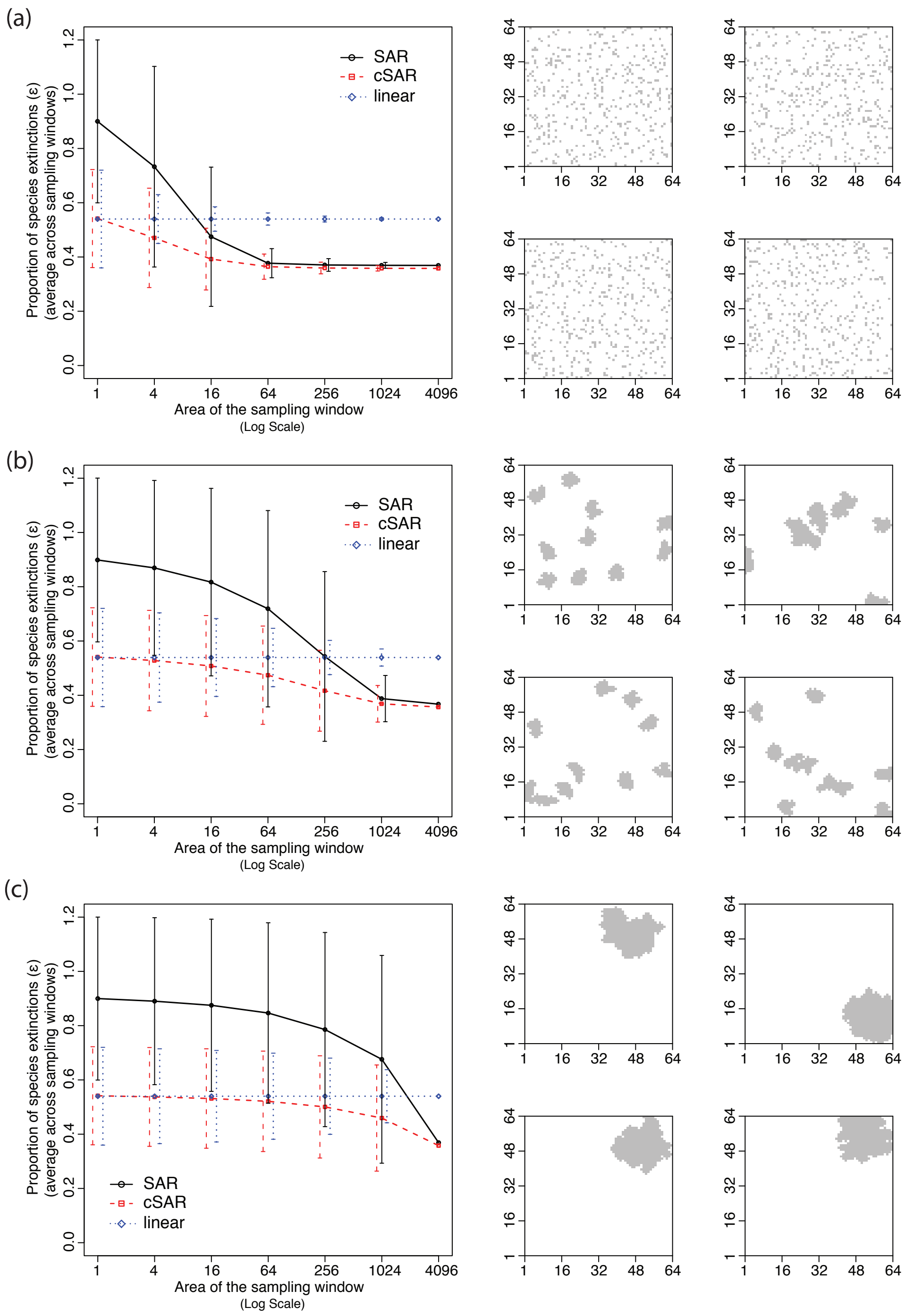

Supplementary Figure S4. Proportion of species extinctions (ε) in the simulated landscape after 90% habitat conversion given by the linear, classic SAR and the countryside SAR (z=0.2; h_1=1, h_2=0.01). When varying **(a-c)** the degree of fragmentation of the landscape after habitat conversion. Right panels show examples of the configuration of a simulated landscape to the different degrees of fragmentation, with white squares corresponding to human-modified habitat and grey squares to the native habitat. For all models, z = 0.2, with h_1=1 for the native habitat and h_2 = 0.01 for the modified habitat. Error bars indicate for each model, the standard deviation of the fraction of species remaining at a given sample grain.

Supplementary Tables

Supplementary Table S1. Complete list of local sensitivities (σ - sigma) values. A total of 730 pairwise comparisons across two taxonomic groups (plants and birds), two broad ecological regions (tropical and temperate), and five different human-modified habitats (annual crops, managed forest, permanent crops, pastures and urban) were retrieved from the literature. Studies (‘Source 2’) were considered if they provided data on species richness on both a native habitat and at least one human-modified habitat. For several studies, more than one possible pairwise comparisons was reported.

Source 1	Source 2	Region	Modified habitat	Taxon	sigma
Chaudhary et al. 2015	GLOBIO	Tropical	Annual Crops	Plants	1.00
Chaudhary et al. 2015	GLOBIO	Temperate	Annual Crops	Plants	1.00
Chaudhary et al. 2015	Shrestha et al. (2010)	Tropical	Annual Crops	Plants	0.98
Chaudhary et al. 2015	Zuo et al. (2008)	Temperate	Annual Crops	Plants	0.98
Chaudhary et al. 2015	Zuo et al. (2008)	Temperate	Annual Crops	Plants	0.98
Chaudhary et al. 2015	Zuo et al. (2008)	Temperate	Annual Crops	Plants	0.98
Chaudhary et al. 2015	Zuo et al. (2008)	Temperate	Annual Crops	Plants	0.97
Chaudhary et al. 2015	Zuo et al. (2008)	Temperate	Annual Crops	Plants	0.97
Chaudhary et al. 2015	Dieleman et al. (2000)	Temperate	Annual Crops	Plants	0.96
Chaudhary et al. 2015	Dieleman et al. (2000)	Temperate	Annual Crops	Plants	0.96
Chaudhary et al. 2015	Zuo et al. (2008)	Temperate	Annual Crops	Plants	0.96
Chaudhary et al. 2015	Zuo et al. (2008)	Temperate	Annual Crops	Plants	0.96
Chaudhary et al. 2015	Zuo et al. (2008)	Temperate	Annual Crops	Plants	0.96
Chaudhary et al. 2015	Zuo et al. (2008)	Temperate	Annual Crops	Plants	0.96
Chaudhary et al. 2015	Zuo et al. (2008)	Temperate	Annual Crops	Plants	0.96
Chaudhary et al. 2015	Zuo et al. (2008)	Temperate	Annual Crops	Plants	0.96
Chaudhary et al. 2015	Zuo et al. (2008)	Temperate	Annual Crops	Plants	0.96
Chaudhary et al. 2015	Zuo et al. (2008)	Temperate	Annual Crops	Plants	0.96
Chaudhary et al. 2015	Kiss et al. (1997)	Temperate	Annual Crops	Plants	0.96
Chaudhary et al. 2015	Zuo et al. (2008)	Temperate	Annual Crops	Plants	0.96
Chaudhary et al. 2015	Zuo et al. (2008)	Temperate	Annual Crops	Plants	0.96
Chaudhary et al. 2015	Huijser et al. (2004)	Temperate	Annual Crops	Plants	0.95
Chaudhary et al. 2015	Huijser et al. (2004)	Temperate	Annual Crops	Plants	0.94
Chaudhary et al. 2015	Huijser et al. (2004)	Temperate	Annual Crops	Plants	0.94
Chaudhary et al. 2015	Zuo et al. (2008)	Temperate	Annual Crops	Plants	0.94
Chaudhary et al. 2015	Zuo et al. (2008)	Temperate	Annual Crops	Plants	0.94
Chaudhary et al. 2015	Swanton et al. (2006)	Temperate	Annual Crops	Plants	0.93
Chaudhary et al. 2015	Swanton et al. (2006)	Temperate	Annual Crops	Plants	0.93
Chaudhary et al. 2015	Swanton et al. (2006)	Temperate	Annual Crops	Plants	0.93

Chaudhary et al. 2015	GLOBIO	Temperate	Annual Crops	Plants	0.93
Chaudhary et al. 2015	Swanton et al. (2006)	Temperate	Annual Crops	Plants	0.92
Chaudhary et al. 2015	Swanton et al. (2006)	Temperate	Annual Crops	Plants	0.92
Chaudhary et al. 2015	Swanton et al. (2006)	Temperate	Annual Crops	Plants	0.92
Chaudhary et al. 2015	Mulugeta et al. (2001)	Temperate	Annual Crops	Plants	0.91
Chaudhary et al. 2015	Caballero-López et al. (2012)	Temperate	Annual Crops	Plants	0.91
Chaudhary et al. 2015	GLOBIO	Temperate	Annual Crops	Plants	0.91
Chaudhary et al. 2015	Légère et al. (2005)	Temperate	Annual Crops	Plants	0.90
Chaudhary et al. 2015	Mulugeta et al. (2001)	Temperate	Annual Crops	Plants	0.90
Chaudhary et al. 2015	GLOBIO	Temperate	Annual Crops	Plants	0.90
Chaudhary et al. 2015	GLOBIO	Temperate	Annual Crops	Plants	0.89
Chaudhary et al. 2015	Légère et al. (2005)	Temperate	Annual Crops	Plants	0.89
Chaudhary et al. 2015	Shrestha et al. (2010)	Tropical	Annual Crops	Plants	0.88
Chaudhary et al. 2015	Mulugeta et al. (2001)	Temperate	Annual Crops	Plants	0.88
Chaudhary et al. 2015	Mulugeta et al. (2001)	Temperate	Annual Crops	Plants	0.88
Chaudhary et al. 2015	GLOBIO	Tropical	Annual Crops	Plants	0.88
Chaudhary et al. 2015	GLOBIO	Temperate	Annual Crops	Plants	0.87
Chaudhary et al. 2015	Légère et al. (2005)	Temperate	Annual Crops	Plants	0.87
Chaudhary et al. 2015	Légère et al. (2005)	Temperate	Annual Crops	Plants	0.87
Chaudhary et al. 2015	Salonen et al. (2001)	Temperate	Annual Crops	Plants	0.87
Chaudhary et al. 2015	Mulugeta et al. (2001)	Temperate	Annual Crops	Plants	0.87
Chaudhary et al. 2015	GLOBIO	Tropical	Annual Crops	Plants	0.85
Chaudhary et al. 2015	Shrestha et al. (2010)	Tropical	Annual Crops	Plants	0.85
Chaudhary et al. 2015	Gillison et al. (2003)	Tropical	Annual Crops	Plants	0.85
Chaudhary et al. 2015	Paine et al. (1996)	Temperate	Annual Crops	Plants	0.85
Chaudhary et al. 2015	Mulugeta et al. (2001)	Temperate	Annual Crops	Plants	0.85
Chaudhary et al. 2015	GLOBIO	Temperate	Annual Crops	Plants	0.84
Chaudhary et al. 2015	Mulugeta et al. (2001)	Temperate	Annual Crops	Plants	0.84
Chaudhary et al. 2015	Hernandez Plaza et al. (2011)	Temperate	Annual Crops	Plants	0.83
Chaudhary et al. 2015	Hernandez Plaza et al. (2011)	Temperate	Annual Crops	Plants	0.82
Chaudhary et al. 2015	Caballero-López et al. (2012)	Temperate	Annual Crops	Plants	0.80
Chaudhary et al. 2015	Hernandez Plaza et al. (2011)	Temperate	Annual Crops	Plants	0.80
Chaudhary et al. 2015	Boutin (2006)	Temperate	Annual Crops	Plants	0.79
Chaudhary et al. 2015	GLOBIO	Tropical	Annual Crops	Plants	0.78
Chaudhary et al. 2015	Caballero-López et al. (2012)	Temperate	Annual Crops	Plants	0.77
Chaudhary et al. 2015	Mulugeta et al. (2001)	Temperate	Annual Crops	Plants	0.76
Chaudhary et al. 2015	Young & Thorne (2004)	Temperate	Annual Crops	Plants	0.76
Chaudhary et al. 2015	GLOBIO	Temperate	Annual Crops	Plants	0.76
Chaudhary et al. 2015	GLOBIO	Temperate	Annual Crops	Plants	0.75
Chaudhary et al. 2015	Gabriel et al. (2005)	Temperate	Annual Crops	Plants	0.75
Chaudhary et al. 2015	Salonen et al. (2001)	Temperate	Annual Crops	Plants	0.74

Chaudhary et al. 2015	Boutin (2006)	Temperate	Annual Crops	Plants	0.72
Chaudhary et al. 2015	Diehl et al. (2012)	Temperate	Annual Crops	Plants	0.72
Chaudhary et al. 2015	GLOBIO	Temperate	Annual Crops	Plants	0.71
Chaudhary et al. 2015	GLOBIO	Temperate	Annual Crops	Plants	0.71
Chaudhary et al. 2015	Cook et al. (2007)	Temperate	Annual Crops	Plants	0.68
Chaudhary et al. 2015	GLOBIO	Temperate	Annual Crops	Plants	0.63
Chaudhary et al. 2015	GLOBIO	Tropical	Annual Crops	Plants	0.62
Chaudhary et al. 2015	Hiltbrunner et al. (2008)	Temperate	Annual Crops	Plants	0.61
Chaudhary et al. 2015	De La Fuente et al. (2010)	Temperate	Annual Crops	Plants	0.61
Chaudhary et al. 2015	GLOBIO	Tropical	Annual Crops	Plants	0.60
Chaudhary et al. 2015	Hyvönen & Salonen (2002)	Temperate	Annual Crops	Plants	0.59
Chaudhary et al. 2015	GLOBIO	Temperate	Annual Crops	Plants	0.58
Chaudhary et al. 2015	De La Fuente et al. (2006)	Temperate	Annual Crops	Plants	0.58
Chaudhary et al. 2015	GLOBIO	Tropical	Annual Crops	Plants	0.56
Chaudhary et al. 2015	GLOBIO	Tropical	Annual Crops	Plants	0.56
Chaudhary et al. 2015	Semere & Slater (2007)	Temperate	Annual Crops	Plants	0.54
Chaudhary et al. 2015	Gardiner et al. (2010)	Temperate	Annual Crops	Plants	0.53
Chaudhary et al. 2015	Hiltbrunner et al. (2008)	Temperate	Annual Crops	Plants	0.52
Chaudhary et al. 2015	Ulber et al. (2009)	Temperate	Annual Crops	Plants	0.52
Chaudhary et al. 2015	Hyvönen & Salonen (2002)	Temperate	Annual Crops	Plants	0.51
Chaudhary et al. 2015	GLOBIO	Tropical	Annual Crops	Plants	0.50
Chaudhary et al. 2015	Hyvönen & Salonen (2002)	Temperate	Annual Crops	Plants	0.50
Chaudhary et al. 2015	GLOBIO	Tropical	Annual Crops	Plants	0.48
Chaudhary et al. 2015	Hiltbrunner et al. (2008)	Temperate	Annual Crops	Plants	0.48
Chaudhary et al. 2015	Hyvönen & Salonen (2002)	Temperate	Annual Crops	Plants	0.48
Chaudhary et al. 2015	GLOBIO	Tropical	Annual Crops	Plants	0.45
Chaudhary et al. 2015	Hiltbrunner et al. (2008)	Temperate	Annual Crops	Plants	0.45
Chaudhary et al. 2015	GLOBIO	Tropical	Annual Crops	Plants	0.43
Chaudhary et al. 2015	Ulber et al. (2009)	Temperate	Annual Crops	Plants	0.41
Chaudhary et al. 2015	GLOBIO	Tropical	Annual Crops	Plants	0.40
Chaudhary et al. 2015	Bátary et al. (2012)	Temperate	Annual Crops	Plants	0.38
Chaudhary et al. 2015	Hyvönen & Salonen (2002)	Temperate	Annual Crops	Plants	0.38
Chaudhary et al. 2015	Roschewitz et al. (2005)	Temperate	Annual Crops	Plants	0.37
Chaudhary et al. 2015	GLOBIO	Tropical	Annual Crops	Plants	0.36
Chaudhary et al. 2015	Hyvönen & Salonen (2002)	Temperate	Annual Crops	Plants	0.35
Chaudhary et al. 2015	GLOBIO	Temperate	Annual Crops	Plants	0.34
Chaudhary et al. 2015	GLOBIO	Temperate	Annual Crops	Plants	0.32
Chaudhary et al. 2015	GLOBIO	Tropical	Annual Crops	Plants	0.28
Chaudhary et al. 2015	GLOBIO	Temperate	Annual Crops	Plants	0.28
Chaudhary et al. 2015	Salonen et al. (2001)	Temperate	Annual Crops	Plants	0.25
Chaudhary et al. 2015	GLOBIO	Temperate	Annual Crops	Plants	0.24

Chaudhary et al. 2015	Salonen et al. (2001)	Temperate	Annual Crops	Plants	0.23
Chaudhary et al. 2015	Semere & Slater (2007)	Temperate	Annual Crops	Plants	0.18
Chaudhary et al. 2015	Salonen et al. (2001)	Temperate	Annual Crops	Plants	0.17
Chaudhary et al. 2015	Roschewitz et al. (2005)	Temperate	Annual Crops	Plants	0.14
Chaudhary et al. 2015	GLOBIO	Temperate	Annual Crops	Plants	0.10
Chaudhary et al. 2015	Roschewitz et al. (2005)	Temperate	Annual Crops	Plants	0.08
Chaudhary et al. 2015	Ulber et al. (2009)	Temperate	Annual Crops	Plants	0.08
Chaudhary et al. 2015	Salonen et al. (2001)	Temperate	Annual Crops	Plants	0.06
Chaudhary et al. 2015	GLOBIO	Tropical	Annual Crops	Plants	0.02
Chaudhary et al. 2015	Salonen et al. (2001)	Temperate	Annual Crops	Plants	0.02
Chaudhary et al. 2015	GLOBIO	Temperate	Annual Crops	Plants	0.00
Chaudhary et al. 2015	Salonen et al. (2001)	Temperate	Annual Crops	Plants	-0.01
Chaudhary et al. 2015	Salonen et al. (2001)	Temperate	Annual Crops	Plants	-0.03
Chaudhary et al. 2015	Salonen et al. (2001)	Temperate	Annual Crops	Plants	-0.04
Chaudhary et al. 2015	Hyvönen et al. (2003)	Temperate	Annual Crops	Plants	-0.04
Chaudhary et al. 2015	Bátary et al. (2012)	Temperate	Annual Crops	Plants	-0.08
Chaudhary et al. 2015	GLOBIO	Temperate	Annual Crops	Plants	-0.08
Chaudhary et al. 2015	Salonen et al. (2001)	Temperate	Annual Crops	Plants	-0.11
Chaudhary et al. 2015	Hald (1999)	Temperate	Annual Crops	Plants	-0.13
Chaudhary et al. 2015	Ulber et al. (2009)	Temperate	Annual Crops	Plants	-0.17
Chaudhary et al. 2015	Salonen et al. (2001)	Temperate	Annual Crops	Plants	-0.19
Chaudhary et al. 2015	GLOBIO	Temperate	Annual Crops	Plants	-0.21
Chaudhary et al. 2015	Hyvönen et al. (2003)	Temperate	Annual Crops	Plants	-0.22
Chaudhary et al. 2015	Salonen et al. (2001)	Temperate	Annual Crops	Plants	-0.27
Chaudhary et al. 2015	GLOBIO	Temperate	Annual Crops	Plants	-0.36
Chaudhary et al. 2015	GLOBIO	Temperate	Annual Crops	Plants	-0.37
Chaudhary et al. 2015	Mulugeta et al. (2001)	Temperate	Annual Crops	Birds	0.85
Chaudhary et al. 2015	Estrada et al. (1997)	Tropical	Annual Crops	Birds	0.83
Chaudhary et al. 2015	GLOBIO	Tropical	Annual Crops	Birds	0.80
Chaudhary et al. 2015	GLOBIO	Tropical	Annual Crops	Birds	0.76
Chaudhary et al. 2015	GLOBIO	Temperate	Annual Crops	Birds	0.75
Chaudhary et al. 2015	Manica et al. (2010)	Tropical	Annual Crops	Birds	0.75
Chaudhary et al. 2015	GLOBIO	Tropical	Annual Crops	Birds	0.73
Chaudhary et al. 2015	Roth et al. (2005)	Temperate	Annual Crops	Birds	0.73
Chaudhary et al. 2015	GLOBIO	Temperate	Annual Crops	Birds	0.70
Chaudhary et al. 2015	GLOBIO	Tropical	Annual Crops	Birds	0.70
Chaudhary et al. 2015	Murray & Best (2003)	Temperate	Annual Crops	Birds	0.68
Chaudhary et al. 2015	Estrada & Coates-Estrada (2005)	Tropical	Annual Crops	Birds	0.67
Chaudhary et al. 2015	Boutin et al. (1999)	Temperate	Annual Crops	Birds	0.63
Chaudhary et al. 2015	GLOBIO	Tropical	Annual Crops	Birds	0.62
Chaudhary et al. 2015	Paine et al. (1996)	Temperate	Annual Crops	Birds	0.62

Chaudhary et al. 2015	Roth et al. (2005)	Temperate	Annual Crops	Birds	0.62
Chaudhary et al. 2015	GLOBIO	Temperate	Annual Crops	Birds	0.61
Chaudhary et al. 2015	Galle et al. (2009)	Temperate	Annual Crops	Birds	0.59
Chaudhary et al. 2015	Boutin et al. (1999)	Temperate	Annual Crops	Birds	0.57
Chaudhary et al. 2015	GLOBIO	Temperate	Annual Crops	Birds	0.54
Chaudhary et al. 2015	GLOBIO	Tropical	Annual Crops	Birds	0.53
Chaudhary et al. 2015	Guerrero et al. (2010)	Temperate	Annual Crops	Birds	0.44
Chaudhary et al. 2015	Galle et al. (2009)	Temperate	Annual Crops	Birds	0.42
Chaudhary et al. 2015	GLOBIO	Tropical	Annual Crops	Birds	0.41
Chaudhary et al. 2015	Galle et al. (2009)	Temperate	Annual Crops	Birds	0.35
Chaudhary et al. 2015	GLOBIO	Temperate	Annual Crops	Birds	0.33
Chaudhary et al. 2015	Bakker & Higgins (2009)	Temperate	Annual Crops	Birds	0.33
Chaudhary et al. 2015	Paine et al. (1996)	Temperate	Annual Crops	Birds	0.31
Chaudhary et al. 2015	GLOBIO	Temperate	Annual Crops	Birds	0.30
Chaudhary et al. 2015	Paine et al. (1996)	Temperate	Annual Crops	Birds	0.23
Chaudhary et al. 2015	GLOBIO	Tropical	Annual Crops	Birds	0.21
Chaudhary et al. 2015	Robertson et al. (2011)	Temperate	Annual Crops	Birds	0.20
Chaudhary et al. 2015	GLOBIO	Tropical	Annual Crops	Birds	0.15
Chaudhary et al. 2015	GLOBIO	Tropical	Annual Crops	Birds	0.13
Chaudhary et al. 2015	GLOBIO	Temperate	Annual Crops	Birds	0.00
Chaudhary et al. 2015	Kovács-Hostyánszki et al. (2011)	Temperate	Annual Crops	Birds	0.00
Chaudhary et al. 2015	GLOBIO	Tropical	Annual Crops	Birds	0.00
Chaudhary et al. 2015	Eggers et al. (2011)	Temperate	Annual Crops	Birds	-0.02
Chaudhary et al. 2015	GLOBIO	Tropical	Annual Crops	Birds	-0.14
Sodhi et al. 2008	Aratrakorn et al. 2006	Tropical	Annual Crops	Birds	0.61
Sodhi et al. 2008	Aratrakorn et al. 2006	Tropical	Annual Crops	Birds	0.60
Sodhi et al. 2008	Kessler et al. 2005	Tropical	Annual Crops	Plants	0.98
Sodhi et al. 2008	Kessler et al. 2005	Tropical	Annual Crops	Plants	0.43
Sodhi et al. 2008	Kessler et al. 2005	Tropical	Annual Crops	Plants	0.99
Sodhi et al. 2008	Kessler et al. 2005	Tropical	Annual Crops	Plants	0.46
Sodhi et al. 2008	Lee et al. 2005	Tropical	Annual Crops	Birds	0.45
Sodhi et al. 2008	Peh et al. 2005	Tropical	Annual Crops	Birds	0.51
Sodhi et al. 2008	Peh et al. 2005	Tropical	Annual Crops	Birds	0.43
Sodhi et al. 2008	Posa & Sodhi 2006	Tropical	Annual Crops	Birds	0.74
Sodhi et al. 2008	Posa & Sodhi 2006	Tropical	Annual Crops	Birds	0.39
Sodhi et al. 2008	Posa & Sodhi 2006	Tropical	Annual Crops	Birds	0.29
Sodhi et al. 2008	Posa & Sodhi 2006	Tropical	Annual Crops	Birds	0.89
Sodhi et al. 2008	Posa & Sodhi 2006	Tropical	Annual Crops	Birds	0.53
Sodhi et al. 2008	Posa & Sodhi 2006	Tropical	Annual Crops	Birds	0.37
Sodhi et al. 2008	Schulze et al. 2004	Tropical	Annual Crops	Birds	0.81
Sodhi et al. 2008	Schulze et al. 2004	Tropical	Annual Crops	Birds	0.78

Sodhi et al. 2008	Schulze et al. 2004	Tropical	Annual Crops	Birds	0.88
Sodhi et al. 2008	Schulze et al. 2004	Tropical	Annual Crops	Birds	0.87
Sodhi et al. 2008	Schulze et al. 2004	Tropical	Annual Crops	Birds	0.94
Sodhi et al. 2008	Schulze et al. 2004	Tropical	Annual Crops	Birds	0.89
Sodhi et al. 2008	Schulze et al. 2004	Tropical	Annual Crops	Plants	0.98
Sodhi et al. 2008	Schulze et al. 2004	Tropical	Annual Crops	Plants	0.87
Sodhi et al. 2008	Schulze et al. 2004	Tropical	Annual Crops	Plants	0.69
Sodhi et al. 2008	Schulze et al. 2004	Tropical	Annual Crops	Plants	0.50
Sodhi et al. 2008	Schulze et al. 2004	Tropical	Annual Crops	Birds	0.81
Sodhi et al. 2008	Schulze et al. 2004	Tropical	Annual Crops	Birds	0.81
Sodhi et al. 2008	Sodhi et al. 2005a	Tropical	Annual Crops	Birds	0.87
Sodhi et al. 2008	Sodhi et al. 2005b	Tropical	Annual Crops	Birds	0.51
Sodhi et al. 2008	Sodhi et al. 2005b	Tropical	Annual Crops	Birds	0.05
Sodhi et al. 2008	Soh et al. 2006	Tropical	Annual Crops	Birds	-0.18
Sodhi et al. 2008	Soh et al. 2006	Tropical	Annual Crops	Birds	0.67
Sodhi et al. 2008	Soh et al. 2006	Tropical	Annual Crops	Birds	0.53
Sodhi et al. 2008	Soh et al. 2006	Tropical	Annual Crops	Birds	0.11
Sodhi et al. 2008	Soh et al. 2006	Tropical	Annual Crops	Birds	0.05
Sodhi et al. 2008	Thiollay & Meyburg 1988	Tropical	Annual Crops	Birds	0.17
Sodhi et al. 2008	Thiollay 1995	Tropical	Annual Crops	Birds	0.62
Sodhi et al. 2008	Thiollay 1995	Tropical	Annual Crops	Birds	0.49
Sodhi et al. 2008	Thiollay 1995	Tropical	Annual Crops	Birds	0.42
Sodhi et al. 2008	Waltert et al. 2004	Tropical	Annual Crops	Birds	-0.71
Sodhi et al. 2008	Waltert et al. 2004	Tropical	Annual Crops	Birds	-0.78
Sodhi et al. 2008	Waltert et al. 2004	Tropical	Annual Crops	Birds	0.53
Sodhi et al. 2008	Waltert et al. 2004	Tropical	Annual Crops	Birds	0.27
Sodhi et al. 2008	Waltert et al. 2005	Tropical	Annual Crops	Birds	0.29
Chaudhary et al. 2015	Paillet et al. 2009	Temperate	Managed Forest	Plants	0.77
Chaudhary et al. 2015	Paillet et al. 2009	Temperate	Managed Forest	Plants	0.76
Chaudhary et al. 2015	Paillet et al. 2009	Temperate	Managed Forest	Plants	0.59
Chaudhary et al. 2015	BDM, 2004	Temperate	Managed Forest	Plants	0.53
Chaudhary et al. 2015	BDM, 2004	Temperate	Managed Forest	Plants	0.53
Chaudhary et al. 2015	Imai (2012)	Tropical	Managed Forest	Plants	0.52
Chaudhary et al. 2015	Putz et al. 2012	Tropical	Managed Forest	Plants	0.50
Chaudhary et al. 2015	GLOBIO	Tropical	Managed Forest	Plants	0.50
Chaudhary et al. 2015	Paillet et al. 2009	Temperate	Managed Forest	Plants	0.50
Chaudhary et al. 2015	BDM, 2004	Temperate	Managed Forest	Plants	0.45
Chaudhary et al. 2015	Adnan&Hölscher (2012)	Tropical	Managed Forest	Plants	0.44
Chaudhary et al. 2015	Gibson et al. 2011	Tropical	Managed Forest	Plants	0.43
Chaudhary et al. 2015	Gibson et al. 2011	Tropical	Managed Forest	Plants	0.42
Chaudhary et al. 2015	Adnan&Hölscher (2012)	Tropical	Managed Forest	Plants	0.39

Chaudhary et al. 2015	BDM, 2004	Temperate	Managed Forest	Plants	0.37
Chaudhary et al. 2015	Putz et al. 2012	Tropical	Managed Forest	Plants	0.34
Chaudhary et al. 2015	Putz et al. 2012	Tropical	Managed Forest	Plants	0.32
Chaudhary et al. 2015	Putz et al. 2012	Tropical	Managed Forest	Plants	0.32
Chaudhary et al. 2015	BDM, 2004	Temperate	Managed Forest	Plants	0.28
Chaudhary et al. 2015	Gibson et al. 2011	Tropical	Managed Forest	Plants	0.26
Chaudhary et al. 2015	Gibson et al. 2011	Tropical	Managed Forest	Plants	0.25
Chaudhary et al. 2015	Gibson et al. 2011	Tropical	Managed Forest	Plants	0.25
Chaudhary et al. 2015	BDM, 2004	Temperate	Managed Forest	Plants	0.22
Chaudhary et al. 2015	Putz et al. 2012	Tropical	Managed Forest	Plants	0.22
Chaudhary et al. 2015	Paillet et al. 2009	Temperate	Managed Forest	Plants	0.22
Chaudhary et al. 2015	Gibson et al. 2011	Tropical	Managed Forest	Plants	0.21
Chaudhary et al. 2015	Paillet et al. 2009	Tropical	Managed Forest	Plants	0.20
Chaudhary et al. 2015	Paillet et al. 2009	Temperate	Managed Forest	Plants	0.20
Chaudhary et al. 2015	BDM, 2004	Temperate	Managed Forest	Plants	0.20
Chaudhary et al. 2015	Paillet et al. 2009	Temperate	Managed Forest	Plants	0.20
Chaudhary et al. 2015	Gibson et al. 2011	Tropical	Managed Forest	Plants	0.18
Chaudhary et al. 2015	Boch (2013)	Temperate	Managed Forest	Plants	0.17
Chaudhary et al. 2015	Rosenvald & Lohmus 2008	Tropical	Managed Forest	Plants	0.17
Chaudhary et al. 2015	Meier et al 1995	Temperate	Managed Forest	Plants	0.16
Chaudhary et al. 2015	Gibson et al. 2011	Tropical	Managed Forest	Plants	0.13
Chaudhary et al. 2015	Clark & Covey 2012	Tropical	Managed Forest	Plants	0.13
Chaudhary et al. 2015	Paillet et al. 2009	Temperate	Managed Forest	Plants	0.11
Chaudhary et al. 2015	Gibson et al. 2011	Tropical	Managed Forest	Plants	0.11
Chaudhary et al. 2015	GLOBIO	Tropical	Managed Forest	Plants	0.11
Chaudhary et al. 2015	Meier et al. (1995)	Temperate	Managed Forest	Plants	0.10
Chaudhary et al. 2015	Putz et al. 2012	Tropical	Managed Forest	Plants	0.10
Chaudhary et al. 2015	Gibson et al. 2011	Tropical	Managed Forest	Plants	0.09
Chaudhary et al. 2015	Paillet et al. 2009	Temperate	Managed Forest	Plants	0.09
Chaudhary et al. 2015	Clark & Covey 2012	Tropical	Managed Forest	Plants	0.08
Chaudhary et al. 2015	GLOBIO	Tropical	Managed Forest	Plants	0.07
Chaudhary et al. 2015	GLOBIO	Tropical	Managed Forest	Plants	0.06
Chaudhary et al. 2015	GLOBIO	Tropical	Managed Forest	Plants	0.06
Chaudhary et al. 2015	Paillet et al. 2009	Temperate	Managed Forest	Plants	0.04
Chaudhary et al. 2015	Imai (2012)	Tropical	Managed Forest	Plants	0.04
Chaudhary et al. 2015	GLOBIO	Tropical	Managed Forest	Plants	0.02
Chaudhary et al. 2015	Gibson et al. 2011	Tropical	Managed Forest	Plants	0.01
Chaudhary et al. 2015	Putz et al. 2012	Tropical	Managed Forest	Plants	0.00
Chaudhary et al. 2015	Paillet et al. 2009	Temperate	Managed Forest	Plants	0.00
Chaudhary et al. 2015	GLOBIO	Tropical	Managed Forest	Plants	0.00
Chaudhary et al. 2015	Paillet et al. 2009	Temperate	Managed Forest	Plants	0.00

Chaudhary et al. 2015	Carreño-Rocabado 2012	Tropical	Managed Forest	Plants	-0.02
Chaudhary et al. 2015	Paillet et al. 2009	Temperate	Managed Forest	Plants	-0.03
Chaudhary et al. 2015	Gibson et al. 2011	Tropical	Managed Forest	Plants	-0.03
Chaudhary et al. 2015	Paillet et al. 2009	Temperate	Managed Forest	Plants	-0.05
Chaudhary et al. 2015	Paillet et al. 2009	Temperate	Managed Forest	Plants	-0.06
Chaudhary et al. 2015	Carreño-Rocabado 2012	Tropical	Managed Forest	Plants	-0.07
Chaudhary et al. 2015	Paillet et al. 2009	Temperate	Managed Forest	Plants	-0.07
Chaudhary et al. 2015	Gibson et al. 2011	Tropical	Managed Forest	Plants	-0.07
Chaudhary et al. 2015	Carreño-Rocabado 2012	Tropical	Managed Forest	Plants	-0.07
Chaudhary et al. 2015	BDM, 2004	Temperate	Managed Forest	Plants	-0.08
Chaudhary et al. 2015	GLOBIO	Tropical	Managed Forest	Plants	-0.08
Chaudhary et al. 2015	BDM, 2004	Temperate	Managed Forest	Plants	-0.09
Chaudhary et al. 2015	Paillet et al. 2009	Temperate	Managed Forest	Plants	-0.11
Chaudhary et al. 2015	Paillet et al. 2009	Temperate	Managed Forest	Plants	-0.14
Chaudhary et al. 2015	Paillet et al. 2009	Temperate	Managed Forest	Plants	-0.14
Chaudhary et al. 2015	BDM, 2004	Temperate	Managed Forest	Plants	-0.16
Chaudhary et al. 2015	Gibson et al. 2011	Tropical	Managed Forest	Plants	-0.16
Chaudhary et al. 2015	GLOBIO	Tropical	Managed Forest	Plants	-0.18
Chaudhary et al. 2015	BDM, 2004	Temperate	Managed Forest	Plants	-0.19
Chaudhary et al. 2015	Boch (2013)	Temperate	Managed Forest	Plants	-0.20
Chaudhary et al. 2015	Durak (2010)	Temperate	Managed Forest	Plants	-0.23
Chaudhary et al. 2015	Paillet et al. 2009	Temperate	Managed Forest	Plants	-0.24
Chaudhary et al. 2015	BDM, 2004	Temperate	Managed Forest	Plants	-0.25
Chaudhary et al. 2015	Putz et al. 2012	Tropical	Managed Forest	Plants	-0.27
Chaudhary et al. 2015	Paillet et al. 2009	Temperate	Managed Forest	Plants	-0.30
Chaudhary et al. 2015	Putz et al. 2012	Temperate	Managed Forest	Birds	0.53
Chaudhary et al. 2015	Putz et al. 2012	Tropical	Managed Forest	Birds	0.43
Chaudhary et al. 2015	Putz et al. 2012	Tropical	Managed Forest	Birds	0.32
Chaudhary et al. 2015	Putz et al. 2012	Tropical	Managed Forest	Birds	0.26
Chaudhary et al. 2015	Putz et al. 2012	Tropical	Managed Forest	Birds	0.25
Chaudhary et al. 2015	Putz et al. 2012	Tropical	Managed Forest	Birds	0.22
Chaudhary et al. 2015	Paillet et al. 2009	Temperate	Managed Forest	Birds	0.21
Chaudhary et al. 2015	Putz et al. 2012	Temperate	Managed Forest	Birds	0.20
Chaudhary et al. 2015	GLOBIO	Temperate	Managed Forest	Birds	0.20
Chaudhary et al. 2015	Paillet et al. 2009	Temperate	Managed Forest	Birds	0.17
Chaudhary et al. 2015	Murphy & Romanuk 2014	Tropical	Managed Forest	Birds	0.15
Chaudhary et al. 2015	Politi (2012)	Tropical	Managed Forest	Birds	0.14
Chaudhary et al. 2015	Clark & Covey 2012	Tropical	Managed Forest	Birds	0.13
Chaudhary et al. 2015	Gibson et al. 2011	Tropical	Managed Forest	Birds	0.12
Chaudhary et al. 2015	Putz et al. 2012	Tropical	Managed Forest	Birds	0.11
Chaudhary et al. 2015	Gibson et al. 2011	Tropical	Managed Forest	Birds	0.11

Chaudhary et al. 2015	Moura (2013)	Tropical	Managed Forest	Birds	0.10
Chaudhary et al. 2015	Putz et al. 2012	Tropical	Managed Forest	Birds	0.09
Chaudhary et al. 2015	Putz et al. 2012	Temperate	Managed Forest	Birds	0.08
Chaudhary et al. 2015	Moura (2013)	Tropical	Managed Forest	Birds	0.08
Chaudhary et al. 2015	Gibson et al. 2011	Tropical	Managed Forest	Birds	0.07
Chaudhary et al. 2015	Edwards (2011)	Tropical	Managed Forest	Birds	0.07
Chaudhary et al. 2015	Gibson et al. 2011	Tropical	Managed Forest	Birds	0.07
Chaudhary et al. 2015	Politi (2012)	Tropical	Managed Forest	Birds	0.07
Chaudhary et al. 2015	Moura (2013)	Tropical	Managed Forest	Birds	0.04
Chaudhary et al. 2015	Edwards (2011)	Tropical	Managed Forest	Birds	0.03
Chaudhary et al. 2015	Moura (2013)	Tropical	Managed Forest	Birds	0.02
Chaudhary et al. 2015	Clark & Covey 2012	Tropical	Managed Forest	Birds	0.01
Chaudhary et al. 2015	Edwards (2011)	Tropical	Managed Forest	Birds	0.00
Chaudhary et al. 2015	Gibson et al. 2011	Tropical	Managed Forest	Birds	-0.01
Chaudhary et al. 2015	Edwards (2011)	Tropical	Managed Forest	Birds	-0.10
Chaudhary et al. 2015	Gibson et al. 2011	Tropical	Managed Forest	Plants	0.86
Chaudhary et al. 2015	Gibson et al. 2011	Tropical	Managed Forest	Plants	0.84
Chaudhary et al. 2015	Clark & Covey 2012	Tropical	Managed Forest	Plants	0.83
Chaudhary et al. 2015	Gibson et al. 2011	Tropical	Managed Forest	Plants	0.82
Chaudhary et al. 2015	GLOBIO	Tropical	Managed Forest	Plants	0.81
Chaudhary et al. 2015	GLOBIO	Tropical	Managed Forest	Plants	0.78
Chaudhary et al. 2015	GLOBIO	Tropical	Managed Forest	Plants	0.76
Chaudhary et al. 2015	Gibson et al. 2011	Temperate	Managed Forest	Plants	0.73
Chaudhary et al. 2015	GLOBIO	Tropical	Managed Forest	Plants	0.73
Chaudhary et al. 2015	Gibson et al. 2011	Tropical	Managed Forest	Plants	0.73
Chaudhary et al. 2015	GLOBIO	Tropical	Managed Forest	Plants	0.69
Chaudhary et al. 2015	GLOBIO	Tropical	Managed Forest	Plants	0.67
Chaudhary et al. 2015	Duguid & Ashton 2013	Temperate	Managed Forest	Plants	0.67
Chaudhary et al. 2015	Gibson et al. 2011	Temperate	Managed Forest	Plants	0.63
Chaudhary et al. 2015	GLOBIO	Tropical	Managed Forest	Plants	0.63
Chaudhary et al. 2015	Clark & Covey 2012	Tropical	Managed Forest	Plants	0.61
Chaudhary et al. 2015	Gibson et al. 2011	Tropical	Managed Forest	Plants	0.60
Chaudhary et al. 2015	Rosenvald & Lohmus 2008	Temperate	Managed Forest	Plants	0.59
Chaudhary et al. 2015	Gibson et al. 2011	Tropical	Managed Forest	Plants	0.59
Chaudhary et al. 2015	Gibson et al. 2011	Tropical	Managed Forest	Plants	0.59
Chaudhary et al. 2015	GLOBIO	Tropical	Managed Forest	Plants	0.58
Chaudhary et al. 2015	Gibson et al. 2011	Temperate	Managed Forest	Plants	0.56
Chaudhary et al. 2015	Gibson et al. 2011	Tropical	Managed Forest	Plants	0.55
Chaudhary et al. 2015	Clark & Covey 2012	Tropical	Managed Forest	Plants	0.54
Chaudhary et al. 2015	Gibson et al. 2011	Tropical	Managed Forest	Plants	0.52
Chaudhary et al. 2015	Pena-Claros, 2003	Tropical	Managed Forest	Plants	0.52

Chaudhary et al. 2015	Gibson et al. 2011	Temperate	Managed Forest	Plants	0.51
Chaudhary et al. 2015	GLOBIO	Tropical	Managed Forest	Plants	0.51
Chaudhary et al. 2015	Gibson et al. 2011	Temperate	Managed Forest	Plants	0.50
Chaudhary et al. 2015	Gibson et al. 2011	Temperate	Managed Forest	Plants	0.50
Chaudhary et al. 2015	Gibson et al. 2011	Temperate	Managed Forest	Plants	0.49
Chaudhary et al. 2015	Clark & Covey 2012	Tropical	Managed Forest	Plants	0.48
Chaudhary et al. 2015	Duguid & Ashton 2013	Temperate	Managed Forest	Plants	0.48
Chaudhary et al. 2015	Gibson et al. 2011	Temperate	Managed Forest	Plants	0.47
Chaudhary et al. 2015	Rosenvald & Lohmus 2008	Temperate	Managed Forest	Plants	0.47
Chaudhary et al. 2015	GLOBIO	Tropical	Managed Forest	Plants	0.47
Chaudhary et al. 2015	Boch (2013)	Temperate	Managed Forest	Plants	0.46
Chaudhary et al. 2015	Duguid & Ashton 2013	Temperate	Managed Forest	Plants	0.46
Chaudhary et al. 2015	Mitchell et al 1997	Temperate	Managed Forest	Plants	0.44
Chaudhary et al. 2015	Gibson et al. 2011	Temperate	Managed Forest	Plants	0.44
Chaudhary et al. 2015	Duguid & Ashton 2013	Temperate	Managed Forest	Plants	0.43
Chaudhary et al. 2015	GLOBIO	Tropical	Managed Forest	Plants	0.43
Chaudhary et al. 2015	Gibson et al. 2011	Tropical	Managed Forest	Plants	0.42
Chaudhary et al. 2015	Paillet et al. 2009	Temperate	Managed Forest	Plants	0.42
Chaudhary et al. 2015	Rosenvald & Lohmus 2008	Temperate	Managed Forest	Plants	0.41
Chaudhary et al. 2015	Clark & Covey 2012	Temperate	Managed Forest	Plants	0.40
Chaudhary et al. 2015	Gibson et al. 2011	Temperate	Managed Forest	Plants	0.40
Chaudhary et al. 2015	Clark & Covey 2012	Tropical	Managed Forest	Plants	0.40
Chaudhary et al. 2015	Gibson et al. 2011	Tropical	Managed Forest	Plants	0.39
Chaudhary et al. 2015	Paillet et al. 2009	Temperate	Managed Forest	Plants	0.38
Chaudhary et al. 2015	Paillet et al. 2009	Temperate	Managed Forest	Plants	0.35
Chaudhary et al. 2015	Rosenvald & Lohmus 2008	Temperate	Managed Forest	Plants	0.35
Chaudhary et al. 2015	Pena-Claros, 2003	Tropical	Managed Forest	Plants	0.31
Chaudhary et al. 2015	Meier et al (2005)	Temperate	Managed Forest	Plants	0.31
Chaudhary et al. 2015	Paillet et al. 2009	Temperate	Managed Forest	Plants	0.31
Chaudhary et al. 2015	Clark & Covey 2012	Tropical	Managed Forest	Plants	0.31
Chaudhary et al. 2015	GLOBIO	Tropical	Managed Forest	Plants	0.30
Chaudhary et al. 2015	Rosenvald & Lohmus 2008	Temperate	Managed Forest	Plants	0.30
Chaudhary et al. 2015	Heinrichs (2010)	Temperate	Managed Forest	Plants	0.29
Chaudhary et al. 2015	Clark & Covey 2012	Tropical	Managed Forest	Plants	0.28
Chaudhary et al. 2015	Meier et al 1995	Temperate	Managed Forest	Plants	0.28
Chaudhary et al. 2015	Pena-Claros, 2003	Tropical	Managed Forest	Plants	0.28
Chaudhary et al. 2015	Meier et al 1995	Temperate	Managed Forest	Plants	0.27
Chaudhary et al. 2015	GLOBIO	Tropical	Managed Forest	Plants	0.27
Chaudhary et al. 2015	Rosenvald & Lohmus 2008	Temperate	Managed Forest	Plants	0.26
Chaudhary et al. 2015	Turner et al. 1994	Tropical	Managed Forest	Plants	0.26
Chaudhary et al. 2015	Meier et al (2005)	Temperate	Managed Forest	Plants	0.25

Chaudhary et al. 2015	Clark & Covey 2012	Tropical	Managed Forest	Plants	0.25
Chaudhary et al. 2015	GLOBIO	Tropical	Managed Forest	Plants	0.22
Chaudhary et al. 2015	Clark & Covey 2012	Tropical	Managed Forest	Plants	0.22
Chaudhary et al. 2015	Lammertink 2004	Tropical	Managed Forest	Plants	0.21
Chaudhary et al. 2015	Rosenvald & Lohmus 2008	Temperate	Managed Forest	Plants	0.21
Chaudhary et al. 2015	Paillet et al. 2009	Temperate	Managed Forest	Plants	0.21
Chaudhary et al. 2015	Duguid & Ashton 2013	Temperate	Managed Forest	Plants	0.20
Chaudhary et al. 2015	Pena-Claros, 2003	Tropical	Managed Forest	Plants	0.19
Chaudhary et al. 2015	GLOBIO	Temperate	Managed Forest	Plants	0.18
Chaudhary et al. 2015	GLOBIO	Temperate	Managed Forest	Plants	0.17
Chaudhary et al. 2015	Boch (2013)	Temperate	Managed Forest	Plants	0.15
Chaudhary et al. 2015	Duguid & Ashton 2013	Temperate	Managed Forest	Plants	0.15
Chaudhary et al. 2015	GLOBIO	Tropical	Managed Forest	Plants	0.14
Chaudhary et al. 2015	Rosenvald & Lohmus 2008	Temperate	Managed Forest	Plants	0.14
Chaudhary et al. 2015	GLOBIO	Tropical	Managed Forest	Plants	0.13
Chaudhary et al. 2015	Boch (2013)	Temperate	Managed Forest	Plants	0.13
Chaudhary et al. 2015	GLOBIO	Tropical	Managed Forest	Plants	0.12
Chaudhary et al. 2015	Clark & Covey 2012	Temperate	Managed Forest	Plants	0.11
Chaudhary et al. 2015	Torras&Saura (2008)	Temperate	Managed Forest	Plants	0.11
Chaudhary et al. 2015	Gibson et al. 2011	Tropical	Managed Forest	Plants	0.10
Chaudhary et al. 2015	Boch (2013)	Temperate	Managed Forest	Plants	0.10
Chaudhary et al. 2015	Clark & Covey 2012	Temperate	Managed Forest	Plants	0.10
Chaudhary et al. 2015	Duguid & Ashton 2013	Temperate	Managed Forest	Plants	0.09
Chaudhary et al. 2015	Clark & Covey 2012	Temperate	Managed Forest	Plants	0.09
Chaudhary et al. 2015	Rosenvald & Lohmus 2008	Temperate	Managed Forest	Plants	0.09
Chaudhary et al. 2015	Paillet et al. 2009	Temperate	Managed Forest	Plants	0.08
Chaudhary et al. 2015	Nagaike (2005)	Temperate	Managed Forest	Plants	0.08
Chaudhary et al. 2015	Paillet et al. 2009	Temperate	Managed Forest	Plants	0.08
Chaudhary et al. 2015	Meier et al (2005)	Temperate	Managed Forest	Plants	0.08
Chaudhary et al. 2015	Duguid & Ashton 2013	Temperate	Managed Forest	Plants	0.07
Chaudhary et al. 2015	Paillet et al. 2009	Temperate	Managed Forest	Plants	0.07
Chaudhary et al. 2015	Gibson et al. 2011	Tropical	Managed Forest	Plants	0.05
Chaudhary et al. 2015	Clark & Covey 2012	Tropical	Managed Forest	Plants	0.04
Chaudhary et al. 2015	Torras&Saura (2008)	Temperate	Managed Forest	Plants	0.04
Chaudhary et al. 2015	Boch (2013)	Temperate	Managed Forest	Plants	0.04
Chaudhary et al. 2015	Paillet et al. 2009	Temperate	Managed Forest	Plants	0.03
Chaudhary et al. 2015	Paillet et al. 2009	Temperate	Managed Forest	Plants	0.02
Chaudhary et al. 2015	Clark & Covey 2012	Temperate	Managed Forest	Plants	0.00
Chaudhary et al. 2015	Johns 1986	Temperate	Managed Forest	Plants	0.00
Chaudhary et al. 2015	Jones et al. 2003	Tropical	Managed Forest	Plants	0.00
Chaudhary et al. 2015	Thiollay & Meyburg 1988	Tropical	Managed Forest	Plants	0.00

Chaudhary et al. 2015	Duguid & Ashton 2013	Temperate	Managed Forest	Plants	0.00
Chaudhary et al. 2015	Duguid & Ashton 2013	Temperate	Managed Forest	Plants	-0.02
Chaudhary et al. 2015	Paillet et al. 2009	Temperate	Managed Forest	Plants	-0.02
Chaudhary et al. 2015	Nagaike (2005)	Temperate	Managed Forest	Plants	-0.05
Chaudhary et al. 2015	Duguid & Ashton 2013	Temperate	Managed Forest	Plants	-0.05
Chaudhary et al. 2015	Heinrichs (2010)	Temperate	Managed Forest	Plants	-0.06
Chaudhary et al. 2015	Heinrichs (2010)	Temperate	Managed Forest	Plants	-0.09
Chaudhary et al. 2015	Paillet et al. 2009	Temperate	Managed Forest	Plants	-0.10
Chaudhary et al. 2015	Paillet et al. 2009	Temperate	Managed Forest	Plants	-0.10
Chaudhary et al. 2015	Duguid & Ashton 2013	Temperate	Managed Forest	Plants	-0.12
Chaudhary et al. 2015	Baral & Katzensteiner 2009	Tropical	Managed Forest	Plants	-0.13
Chaudhary et al. 2015	Clark & Covey 2012	Tropical	Managed Forest	Plants	-0.13
Chaudhary et al. 2015	Paillet et al. 2009	Temperate	Managed Forest	Plants	-0.14
Chaudhary et al. 2015	Paillet et al. 2009	Temperate	Managed Forest	Plants	-0.14
Chaudhary et al. 2015	Rosenvald & Lohmus 2008	Temperate	Managed Forest	Plants	-0.15
Chaudhary et al. 2015	Paillet et al. 2009	Temperate	Managed Forest	Plants	-0.16
Chaudhary et al. 2015	Clark & Covey 2012	Tropical	Managed Forest	Plants	-0.17
Chaudhary et al. 2015	Paillet et al. 2009	Temperate	Managed Forest	Plants	-0.17
Chaudhary et al. 2015	Paillet et al. 2009	Temperate	Managed Forest	Plants	-0.18
Chaudhary et al. 2015	Duguid & Ashton 2013	Temperate	Managed Forest	Plants	-0.18
Chaudhary et al. 2015	Rosenvald & Lohmus 2008	Temperate	Managed Forest	Plants	-0.18
Chaudhary et al. 2015	Duguid & Ashton 2013	Temperate	Managed Forest	Plants	-0.18
Chaudhary et al. 2015	Rosenvald & Lohmus 2008	Temperate	Managed Forest	Plants	-0.20
Chaudhary et al. 2015	GLOBIO	Tropical	Managed Forest	Birds	0.93
Chaudhary et al. 2015	Gibson et al. 2011	Tropical	Managed Forest	Birds	0.88
Chaudhary et al. 2015	Gibson et al. 2011	Tropical	Managed Forest	Birds	0.87
Chaudhary et al. 2015	Moura (2013)	Tropical	Managed Forest	Birds	0.73
Chaudhary et al. 2015	Rosenvald & Lohmus 2008	Temperate	Managed Forest	Birds	0.72
Chaudhary et al. 2015	Rosenvald & Lohmus 2008	Temperate	Managed Forest	Birds	0.69
Chaudhary et al. 2015	Moura (2013)	Tropical	Managed Forest	Birds	0.69
Chaudhary et al. 2015	Rosenvald & Lohmus 2008	Temperate	Managed Forest	Birds	0.63
Chaudhary et al. 2015	Watson 2004	Tropical	Managed Forest	Birds	0.58
Chaudhary et al. 2015	GLOBIO	Temperate	Managed Forest	Birds	0.58
Chaudhary et al. 2015	Rosenvald & Lohmus 2008	Temperate	Managed Forest	Birds	0.56
Chaudhary et al. 2015	Gibson et al. 2011	Tropical	Managed Forest	Birds	0.52
Chaudhary et al. 2015	Gibson et al. 2011	Tropical	Managed Forest	Birds	0.51
Chaudhary et al. 2015	Rosenvald & Lohmus 2008	Temperate	Managed Forest	Birds	0.51
Chaudhary et al. 2015	Moura (2013)	Tropical	Managed Forest	Birds	0.51
Chaudhary et al. 2015	Rosenvald & Lohmus 2008	Temperate	Managed Forest	Birds	0.49
Chaudhary et al. 2015	GLOBIO	Temperate	Managed Forest	Birds	0.48
Chaudhary et al. 2015	Rosenvald & Lohmus 2008	Temperate	Managed Forest	Birds	0.48

Chaudhary et al. 2015	GLOBIO	Tropical	Managed Forest	Birds	0.48
Chaudhary et al. 2015	Gibson et al. 2011	Tropical	Managed Forest	Birds	0.47
Chaudhary et al. 2015	Gibson et al. 2011	Tropical	Managed Forest	Birds	0.41
Chaudhary et al. 2015	Gibson et al. 2011	Tropical	Managed Forest	Birds	0.39
Chaudhary et al. 2015	Round & Brockelman 1998	Tropical	Managed Forest	Birds	0.39
Chaudhary et al. 2015	Rosenvald & Lohmus 2008	Temperate	Managed Forest	Birds	0.39
Chaudhary et al. 2015	Rosenvald & Lohmus 2008	Temperate	Managed Forest	Birds	0.35
Chaudhary et al. 2015	Rosenvald & Lohmus 2008	Temperate	Managed Forest	Birds	0.33
Chaudhary et al. 2015	Gibson et al. 2011	Tropical	Managed Forest	Birds	0.29
Chaudhary et al. 2015	Moura (2013)	Tropical	Managed Forest	Birds	0.28
Chaudhary et al. 2015	Gibson et al. 2011	Tropical	Managed Forest	Birds	0.27
Chaudhary et al. 2015	Rosenvald & Lohmus 2008	Temperate	Managed Forest	Birds	0.27
Chaudhary et al. 2015	Putz et al. 2012	Tropical	Managed Forest	Birds	0.25
Chaudhary et al. 2015	Rosenvald & Lohmus 2008	Temperate	Managed Forest	Birds	0.19
Chaudhary et al. 2015	Paillet et al. 2009	Temperate	Managed Forest	Birds	0.17
Chaudhary et al. 2015	Boch (2013)	Temperate	Managed Forest	Birds	0.09
Chaudhary et al. 2015	Rosenvald & Lohmus 2008	Temperate	Managed Forest	Birds	0.08
Chaudhary et al. 2015	Paillet et al. 2009	Temperate	Managed Forest	Birds	0.06
Chaudhary et al. 2015	GLOBIO	Tropical	Managed Forest	Birds	0.01
Chaudhary et al. 2015	GLOBIO	Tropical	Managed Forest	Birds	-0.08
Chaudhary et al. 2015	Rosenvald & Lohmus 2008	Temperate	Managed Forest	Birds	-0.12
Chaudhary et al. 2015	Rosenvald & Lohmus 2008	Temperate	Managed Forest	Birds	-0.14
Sodhi et al. 2008	Brearley et al. 2004	Tropical	Managed Forest	Plants	0.35
Sodhi et al. 2008	Castelletta et al. 2000	Tropical	Managed Forest	Birds	0.67
Sodhi et al. 2008	Castelletta et al. 2000	Tropical	Managed Forest	Birds	0.32
Sodhi et al. 2008	Diamond et al. 1987	Tropical	Managed Forest	Birds	0.32
Sodhi et al. 2008	Clark & Covey 2012	Tropical	Managed Forest	Birds	0.00
Sodhi et al. 2008	Johns 1986	Tropical	Managed Forest	Birds	0.41
Sodhi et al. 2008	Johns 1986	Tropical	Managed Forest	Birds	0.29
Sodhi et al. 2008	Johns 1986	Tropical	Managed Forest	Birds	0.24
Sodhi et al. 2008	Johns 1986	Tropical	Managed Forest	Birds	0.02
Sodhi et al. 2008	Johns 1986	Tropical	Managed Forest	Birds	0.25
Sodhi et al. 2008	Johns 1986	Tropical	Managed Forest	Birds	0.23
Sodhi et al. 2008	Johns 1986	Tropical	Managed Forest	Birds	0.30
Sodhi et al. 2008	Johns 1986	Tropical	Managed Forest	Birds	0.26
Sodhi et al. 2008	Johns 1989	Tropical	Managed Forest	Birds	0.28
Sodhi et al. 2008	Johns 1989	Tropical	Managed Forest	Birds	0.17
Sodhi et al. 2008	Johns 1996	Tropical	Managed Forest	Birds	-0.37
Sodhi et al. 2008	Johns 1996	Tropical	Managed Forest	Birds	-0.51
Sodhi et al. 2008	Clark & Covey 2012	Tropical	Managed Forest	Birds	0.00
Sodhi et al. 2008	Jones et al. 2003	Tropical	Managed Forest	Birds	-0.20

Sodhi et al. 2008	Jones et al. 2003	Tropical	Managed Forest	Birds	-0.04
Sodhi et al. 2008	Kessler et al. 2005	Tropical	Managed Forest	Plants	0.77
Sodhi et al. 2008	Kessler et al. 2005	Tropical	Managed Forest	Plants	0.82
Sodhi et al. 2008	Lambert & Collar 2002	Tropical	Managed Forest	Birds	0.30
Sodhi et al. 2008	Lambert & Collar 2002	Tropical	Managed Forest	Birds	0.09
Sodhi et al. 2008	Meier et al (2005)	Tropical	Managed Forest	Birds	0.00
Sodhi et al. 2008	Lambert 1992	Tropical	Managed Forest	Birds	0.07
Sodhi et al. 2008	Lambert 1992	Tropical	Managed Forest	Birds	0.17
Sodhi et al. 2008	Lambert 1992	Tropical	Managed Forest	Birds	0.04
Sodhi et al. 2008	Baral & Katzensteiner 2009	Tropical	Managed Forest	Birds	0.21
Sodhi et al. 2008	Lammertink 2004	Tropical	Managed Forest	Birds	0.21
Sodhi et al. 2008	Lammertink 2004	Tropical	Managed Forest	Birds	0.14
Sodhi et al. 2008	Lammertink 2004	Tropical	Managed Forest	Birds	0.14
Sodhi et al. 2008	Lammertink 2004	Tropical	Managed Forest	Birds	0.07
Sodhi et al. 2008	Lammertink 2004	Tropical	Managed Forest	Birds	0.07
Sodhi et al. 2008	Lammertink 2004	Tropical	Managed Forest	Birds	0.07
Sodhi et al. 2008	Lee et al. 2005	Tropical	Managed Forest	Birds	0.10
Sodhi et al. 2008	Marsden 1998	Tropical	Managed Forest	Birds	0.25
Sodhi et al. 2008	Marsden 1998	Tropical	Managed Forest	Birds	0.22
Sodhi et al. 2008	Pattanavibool & Dearden 2002	Tropical	Managed Forest	Birds	0.15
Sodhi et al. 2008	Pattanavibool & Dearden 2002	Tropical	Managed Forest	Birds	0.25
Sodhi et al. 2008	Peh et al. 2005	Tropical	Managed Forest	Birds	0.11
Sodhi et al. 2008	Peh et al. 2005	Tropical	Managed Forest	Birds	0.08
Sodhi et al. 2008	Posa & Sodhi 2006	Tropical	Managed Forest	Birds	-0.08
Sodhi et al. 2008	Posa & Sodhi 2006	Tropical	Managed Forest	Birds	-0.05
Sodhi et al. 2008	Round & Brockelman 1998	Tropical	Managed Forest	Birds	0.39
Sodhi et al. 2008	Round & Brockelman 1998	Tropical	Managed Forest	Birds	0.01
Sodhi et al. 2008	Schulze et al. 2004	Tropical	Managed Forest	Birds	0.46
Sodhi et al. 2008	Schulze et al. 2004	Tropical	Managed Forest	Birds	0.24
Sodhi et al. 2008	Schulze et al. 2004	Tropical	Managed Forest	Birds	0.55
Sodhi et al. 2008	Schulze et al. 2004	Tropical	Managed Forest	Plants	0.67
Sodhi et al. 2008	Schulze et al. 2004	Tropical	Managed Forest	Plants	-0.04
Sodhi et al. 2008	Schulze et al. 2004	Tropical	Managed Forest	Plants	-0.72
Sodhi et al. 2008	Schulze et al. 2004	Tropical	Managed Forest	Birds	0.21
Sodhi et al. 2008	Silk et al. 2002	Tropical	Managed Forest	Plants	0.42
Sodhi et al. 2008	Sodhi 2002	Tropical	Managed Forest	Birds	0.67
Sodhi et al. 2008	Sodhi et al. 2005a	Tropical	Managed Forest	Birds	0.76
Sodhi et al. 2008	Sodhi et al. 2005a	Tropical	Managed Forest	Birds	0.69
Sodhi et al. 2008	Sodhi et al. 2005a	Tropical	Managed Forest	Birds	0.60
Sodhi et al. 2008	Sodhi et al. 2005b	Tropical	Managed Forest	Birds	0.02
Sodhi et al. 2008	Soh et al. 2006	Tropical	Managed Forest	Birds	-0.05

Sodhi et al. 2008	Soh et al. 2006	Tropical	Managed Forest	Birds	-0.34
Sodhi et al. 2008	Styring & Ickes 2001	Tropical	Managed Forest	Birds	0.27
Sodhi et al. 2008	Lambert 1992	Tropical	Managed Forest	Birds	0.00
Sodhi et al. 2008	Thiollay & Meyburg 1988	Tropical	Managed Forest	Birds	-0.67
Sodhi et al. 2008	Turner et al. 1994	Tropical	Managed Forest	Plants	0.39
Sodhi et al. 2008	Turner et al. 1994	Tropical	Managed Forest	Plants	0.05
Sodhi et al. 2008	Turner et al. 1994	Tropical	Managed Forest	Plants	0.29
Sodhi et al. 2008	Clark & Covey 2012	Tropical	Managed Forest	Plants	0.26
Sodhi et al. 2008	Turner et al. 1997	Tropical	Managed Forest	Plants	0.40
Sodhi et al. 2008	Turner et al. 1997	Tropical	Managed Forest	Plants	0.53
Sodhi et al. 2008	Turner et al. 1997	Tropical	Managed Forest	Plants	0.38
Sodhi et al. 2008	Waltert et al. 2005	Tropical	Managed Forest	Birds	0.33
Sodhi et al. 2008	Wong 1986	Tropical	Managed Forest	Birds	0.12
Chaudhary et al. 2015	GLOBIO	Tropical	Pasture	Plants	0.89
Chaudhary et al. 2015	GLOBIO	Tropical	Pasture	Plants	0.86
Chaudhary et al. 2015	GLOBIO	Tropical	Pasture	Plants	0.82
Chaudhary et al. 2015	GLOBIO	Temperate	Pasture	Plants	0.81
Chaudhary et al. 2015	GLOBIO	Temperate	Pasture	Plants	0.80
Chaudhary et al. 2015	GLOBIO	Temperate	Pasture	Plants	0.78
Chaudhary et al. 2015	GLOBIO	Temperate	Pasture	Plants	0.77
Chaudhary et al. 2015	GLOBIO	Temperate	Pasture	Plants	0.75
Chaudhary et al. 2015	GLOBIO	Temperate	Pasture	Plants	0.74
Chaudhary et al. 2015	GLOBIO	Temperate	Pasture	Plants	0.69
Chaudhary et al. 2015	GLOBIO	Temperate	Pasture	Plants	0.69
Chaudhary et al. 2015	GLOBIO	Tropical	Pasture	Plants	0.68
Chaudhary et al. 2015	GLOBIO	Temperate	Pasture	Plants	0.68
Chaudhary et al. 2015	GLOBIO	Temperate	Pasture	Plants	0.67
Chaudhary et al. 2015	GLOBIO	Tropical	Pasture	Plants	0.66
Chaudhary et al. 2015	GLOBIO	Temperate	Pasture	Plants	0.64
Chaudhary et al. 2015	GLOBIO	Temperate	Pasture	Plants	0.57
Chaudhary et al. 2015	GLOBIO	Tropical	Pasture	Plants	0.56
Chaudhary et al. 2015	GLOBIO	Temperate	Pasture	Plants	0.42
Chaudhary et al. 2015	GLOBIO	Temperate	Pasture	Plants	0.42
Chaudhary et al. 2015	GLOBIO	Temperate	Pasture	Plants	0.42
Chaudhary et al. 2015	GLOBIO	Temperate	Pasture	Plants	0.41
Chaudhary et al. 2015	GLOBIO	Temperate	Pasture	Plants	0.41
Chaudhary et al. 2015	GLOBIO	Tropical	Pasture	Plants	0.40
Chaudhary et al. 2015	GLOBIO	Temperate	Pasture	Plants	0.40
Chaudhary et al. 2015	GLOBIO	Temperate	Pasture	Plants	0.39
Chaudhary et al. 2015	GLOBIO	Temperate	Pasture	Plants	0.37
Chaudhary et al. 2015	GLOBIO	Temperate	Pasture	Plants	0.36

Chaudhary et al. 2015	GLOBIO	Tropical	Pasture	Plants	0.34
Chaudhary et al. 2015	GLOBIO	Temperate	Pasture	Plants	0.34
Chaudhary et al. 2015	GLOBIO	Temperate	Pasture	Plants	0.33
Chaudhary et al. 2015	GLOBIO	Temperate	Pasture	Plants	0.32
Chaudhary et al. 2015	GLOBIO	Tropical	Pasture	Plants	0.30
Chaudhary et al. 2015	GLOBIO	Temperate	Pasture	Plants	0.29
Chaudhary et al. 2015	GLOBIO	Temperate	Pasture	Plants	0.29
Chaudhary et al. 2015	GLOBIO	Tropical	Pasture	Plants	0.28
Chaudhary et al. 2015	GLOBIO	Temperate	Pasture	Plants	0.28
Chaudhary et al. 2015	GLOBIO	Temperate	Pasture	Plants	0.24
Chaudhary et al. 2015	GLOBIO	Temperate	Pasture	Plants	0.24
Chaudhary et al. 2015	GLOBIO	Temperate	Pasture	Plants	0.22
Chaudhary et al. 2015	GLOBIO	Temperate	Pasture	Plants	0.14
Chaudhary et al. 2015	GLOBIO	Temperate	Pasture	Plants	0.09
Chaudhary et al. 2015	GLOBIO	Temperate	Pasture	Plants	0.07
Chaudhary et al. 2015	GLOBIO	Temperate	Pasture	Plants	0.05
Chaudhary et al. 2015	GLOBIO	Temperate	Pasture	Plants	0.00
Chaudhary et al. 2015	GLOBIO	Temperate	Pasture	Plants	0.00
Chaudhary et al. 2015	GLOBIO	Temperate	Pasture	Plants	-0.01
Chaudhary et al. 2015	GLOBIO	Temperate	Pasture	Plants	-0.03
Chaudhary et al. 2015	GLOBIO	Temperate	Pasture	Plants	-0.05
Chaudhary et al. 2015	GLOBIO	Temperate	Pasture	Plants	-0.09
Chaudhary et al. 2015	GLOBIO	Temperate	Pasture	Plants	-0.10
Chaudhary et al. 2015	GLOBIO	Temperate	Pasture	Plants	-0.14
Chaudhary et al. 2015	GLOBIO	Temperate	Pasture	Plants	-0.20
Chaudhary et al. 2015	GLOBIO	Temperate	Pasture	Plants	-0.21
Chaudhary et al. 2015	GLOBIO	Temperate	Pasture	Plants	-0.23
Chaudhary et al. 2015	GLOBIO	Temperate	Pasture	Plants	-0.35
Chaudhary et al. 2015	GLOBIO	Temperate	Pasture	Plants	-0.38
Chaudhary et al. 2015	GLOBIO	Temperate	Pasture	Plants	-0.45
Chaudhary et al. 2015	GLOBIO	Temperate	Pasture	Plants	-0.48
Chaudhary et al. 2015	GLOBIO	Tropical	Pasture	Birds	0.93
Chaudhary et al. 2015	GLOBIO	Tropical	Pasture	Birds	0.10
Chaudhary et al. 2015	GLOBIO	Temperate	Pasture	Birds	0.17
Chaudhary et al. 2015	GLOBIO	Temperate	Pasture	Birds	-0.08
Chaudhary et al. 2015	GLOBIO	Tropical	Pasture	Birds	0.57
Chaudhary et al. 2015	GLOBIO	Temperate	Pasture	Birds	-0.50
Chaudhary et al. 2015	GLOBIO	Temperate	Pasture	Birds	-0.67
Chaudhary et al. 2015	GLOBIO	Temperate	Pasture	Birds	-0.61
Chaudhary et al. 2015	GLOBIO	Tropical	Pasture	Birds	0.43
Chaudhary et al. 2015	GLOBIO	Temperate	Pasture	Birds	0.60

Chaudhary et al. 2015	GLOBIO	Temperate	Pasture	Birds	0.47
Chaudhary et al. 2015	GLOBIO	Tropical	Pasture	Birds	0.24
Chaudhary et al. 2015	GLOBIO	Temperate	Pasture	Birds	0.46
Chaudhary et al. 2015	GLOBIO	Tropical	Pasture	Birds	0.04
Chaudhary et al. 2015	GLOBIO	Tropical	Permanent Crops	Plants	0.96
Chaudhary et al. 2015	BDM, 2004	Temperate	Permanent Crops	Plants	0.90
Chaudhary et al. 2015	GLOBIO	Tropical	Permanent Crops	Plants	0.90
Chaudhary et al. 2015	BDM, 2004	Temperate	Permanent Crops	Plants	0.89
Chaudhary et al. 2015	BDM, 2004	Temperate	Permanent Crops	Plants	0.85
Chaudhary et al. 2015	GLOBIO	Tropical	Permanent Crops	Plants	0.85
Chaudhary et al. 2015	GLOBIO	Tropical	Permanent Crops	Plants	0.79
Chaudhary et al. 2015	GLOBIO	Tropical	Permanent Crops	Plants	0.79
Chaudhary et al. 2015	GLOBIO	Tropical	Permanent Crops	Plants	0.75
Chaudhary et al. 2015	Gibson et al. 2011	Tropical	Permanent Crops	Plants	0.68
Chaudhary et al. 2015	Hundera 2013	Temperate	Permanent Crops	Plants	0.59
Chaudhary et al. 2015	Gibson et al. 2011	Tropical	Permanent Crops	Plants	0.58
Chaudhary et al. 2015	BDM, 2004	Temperate	Permanent Crops	Plants	0.56
Chaudhary et al. 2015	BDM, 2004	Temperate	Permanent Crops	Plants	0.51
Chaudhary et al. 2015	Gibson et al. 2011	Tropical	Permanent Crops	Plants	0.45
Chaudhary et al. 2015	Beukema (2007)	Tropical	Permanent Crops	Plants	0.40
Chaudhary et al. 2015	Gibson et al. 2011	Tropical	Permanent Crops	Plants	0.38
Chaudhary et al. 2015	GLOBIO	Tropical	Permanent Crops	Plants	0.35
Chaudhary et al. 2015	GLOBIO	Temperate	Permanent Crops	Plants	0.30
Chaudhary et al. 2015	GLOBIO	Tropical	Permanent Crops	Plants	0.28
Chaudhary et al. 2015	Gibson et al. 2011	Tropical	Permanent Crops	Plants	0.25
Chaudhary et al. 2015	Hundera 2013	Temperate	Permanent Crops	Plants	0.24
Chaudhary et al. 2015	Gibson et al. 2011	Tropical	Permanent Crops	Plants	0.24
Chaudhary et al. 2015	Gibson et al. 2011	Tropical	Permanent Crops	Plants	0.21
Chaudhary et al. 2015	GLOBIO	Tropical	Permanent Crops	Plants	0.09
Chaudhary et al. 2015	GLOBIO	Tropical	Permanent Crops	Plants	-0.03
Chaudhary et al. 2015	BDM, 2004	Temperate	Permanent Crops	Plants	-0.05
Chaudhary et al. 2015	Gibson et al. 2011	Tropical	Permanent Crops	Birds	0.93
Chaudhary et al. 2015	Najera & Simonetti (2010)	Tropical	Permanent Crops	Birds	0.92
Chaudhary et al. 2015	Najera & Simonetti (2010)	Tropical	Permanent Crops	Birds	0.86
Chaudhary et al. 2015	Sheldon & Styring (2011)	Tropical	Permanent Crops	Birds	0.79
Chaudhary et al. 2015	GLOBIO	Tropical	Permanent Crops	Birds	0.76
Chaudhary et al. 2015	Koh & Wilcove (2008)	Tropical	Permanent Crops	Birds	0.75
Chaudhary et al. 2015	Koh & Wilcove (2008)	Tropical	Permanent Crops	Birds	0.75
Chaudhary et al. 2015	Danielsen & Heegaard (1995)	Tropical	Permanent Crops	Birds	0.75
Chaudhary et al. 2015	GLOBIO	Tropical	Permanent Crops	Birds	0.73
Chaudhary et al. 2015	GLOBIO	Tropical	Permanent Crops	Birds	0.71

Chaudhary et al. 2015	Sheldon & Styring (2011)	Tropical	Permanent Crops	Birds	0.66
Chaudhary et al. 2015	Azhar et al. (2011)	Tropical	Permanent Crops	Birds	0.65
Chaudhary et al. 2015	Aratrakorn et al. (2006)	Tropical	Permanent Crops	Birds	0.62
Chaudhary et al. 2015	Murphy & Romanuk 2014	Tropical	Permanent Crops	Birds	0.61
Chaudhary et al. 2015	GLOBIO	Tropical	Permanent Crops	Birds	0.61
Chaudhary et al. 2015	Koh (2008)	Tropical	Permanent Crops	Birds	0.60
Chaudhary et al. 2015	Murphy & Romanuk 2014	Tropical	Permanent Crops	Birds	0.60
Chaudhary et al. 2015	GLOBIO	Tropical	Permanent Crops	Birds	0.57
Chaudhary et al. 2015		Tropical	Permanent Crops	Birds	0.54
Chaudhary et al. 2015	Gibson et al. 2011	Tropical	Permanent Crops	Birds	0.54
Chaudhary et al. 2015	GLOBIO	Tropical	Permanent Crops	Birds	0.54
Chaudhary et al. 2015	GLOBIO	Tropical	Permanent Crops	Birds	0.46
Chaudhary et al. 2015		Tropical	Permanent Crops	Birds	0.41
Chaudhary et al. 2015	GLOBIO	Tropical	Permanent Crops	Birds	0.39
Chaudhary et al. 2015	GLOBIO	Tropical	Permanent Crops	Birds	0.36
Chaudhary et al. 2015	Azhar et al. (2011)	Tropical	Permanent Crops	Birds	0.33
Chaudhary et al. 2015	GLOBIO	Tropical	Permanent Crops	Birds	0.31
Chaudhary et al. 2015	GLOBIO	Tropical	Permanent Crops	Birds	0.29
Chaudhary et al. 2015	GLOBIO	Tropical	Permanent Crops	Birds	0.05
Chaudhary et al. 2015	GLOBIO	Tropical	Permanent Crops	Birds	-0.10
Chaudhary et al. 2015	GLOBIO	Temperate	Urban	Birds	0.67
Chaudhary et al. 2015	GLOBIO	Tropical	Urban	Birds	0.54
Chaudhary et al. 2015	GLOBIO	Temperate	Urban	Birds	0.24
Chaudhary et al. 2015	GLOBIO	Temperate	Urban	Plants	0.80
Chaudhary et al. 2015	GLOBIO	Temperate	Urban	Plants	0.75
Chaudhary et al. 2015	GLOBIO	Temperate	Urban	Plants	0.73
Chaudhary et al. 2015	GLOBIO	Temperate	Urban	Plants	0.71
Chaudhary et al. 2015	GLOBIO	Temperate	Urban	Plants	0.67
Chaudhary et al. 2015	GLOBIO	Temperate	Urban	Plants	0.64
Chaudhary et al. 2015	GLOBIO	Temperate	Urban	Plants	0.62
Chaudhary et al. 2015	GLOBIO	Temperate	Urban	Plants	0.59
Chaudhary et al. 2015	GLOBIO	Temperate	Urban	Plants	0.58
Chaudhary et al. 2015	GLOBIO	Temperate	Urban	Plants	0.58
Chaudhary et al. 2015	GLOBIO	Temperate	Urban	Plants	0.56
Chaudhary et al. 2015	GLOBIO	Temperate	Urban	Plants	0.50
Chaudhary et al. 2015	GLOBIO	Temperate	Urban	Plants	0.46
Chaudhary et al. 2015	GLOBIO	Temperate	Urban	Plants	0.45
Chaudhary et al. 2015	GLOBIO	Temperate	Urban	Plants	0.36
Chaudhary et al. 2015	GLOBIO	Temperate	Urban	Plants	0.29
Chaudhary et al. 2015	GLOBIO	Temperate	Urban	Plants	0.22
Chaudhary et al. 2015	GLOBIO	Temperate	Urban	Plants	0.09

Chaudhary et al. 2015	GLOBIO	Temperate	Urban	Plants	0.00
Chaudhary et al. 2015	GLOBIO	Temperate	Urban	Plants	-0.01
Chaudhary et al. 2015	GLOBIO	Temperate	Urban	Plants	-0.05
Chaudhary et al. 2015	GLOBIO	Temperate	Urban	Plants	-0.18

Supplementary Table S2. Average affinity values (h's) for the different habitats and regions of the world.

	Tropical	Temperate
Annual crops	0.0229	0.0150
Permanent crops	0.0206	0.0219
Pastures	0.0294	0.2999
Managed Forest	0.2365	0.3584
Urban	0.0209	0.0610
